# Supplementary material for: Length of stay following percutaneous left atrial appendage occlusion: Data from the prospective, multicenter Amplatzer Amulet Occluder Observational Study
Source: PLoS One. 2021 Aug 10;16(8):e0255721. doi: 10.1371/journal.pone.0255721 (PMC8354446; doi:10.1371/journal.pone.0255721)
Supplement: S1 File — (PDF) [file pone.0255721.s002.pdf]

	Clinical Investigation Plan Cover Page

NCT Number: 02447081
AMPLATZER Amulet Observational PMS AMPLATZER™
Amulet™ Observational Post-Market Study
Study Document No: SJM-CIP-10053
Version A
Document Date: 21-APR-2015

Sponsor St. Jude Medical, Inc.
5050 Nathan Lane North
Plymouth, MN 55442
USA

AMPLATZER™ Amulet™ Observational Post-Market Study

Clinical Protocol

Sponsor: St. Jude Medical, Inc. (SJM)
5050 Nathan Lane North
Plymouth, MN 55442
USA
Main +1 6517565400
Fax +1 8772572381

Principal Investigator

SIGNATURE PAGE

AMPLATZER™ Amulet™ Observational Post-Market Study

Study Document No: SJM-CIP-10053 Version A

I have read and agree to adhere to the clinical protocol (CP), including the Investigator's Brochure (**Appendix D**), and all regulatory requirements applicable in conducting this clinical study.

Principal Investigator

Printed name: ____

Signature: ____

Date: ____

<u>Coordinating Investigator/ National Investigator/ Medical Advisor</u>

SIGNATURE PAGE

AMPLATZER™ Amulet™ Observational Post-Market Study

Study Document No: SJM-CIP-10053 Version A

I have read and agree to adhere to the clinical protocol (CP), including the Investigator's Brochure (**Appendix D**), and all regulatory requirements applicable in conducting this clinical study.

<u>Coordinating Investigator/ National Investigator/ Medical Advisor</u>

Printed name: ____

Signature: ____

Date: ____

Table of Contents

1. Synopsis 7
1.1 Study Contacts 10
1.2 Certificate History 11
2. Background and Justification 12
3. Risks and Benefits 14
3.1 Anticipated Clinical Benefits 14
3.2 Anticipated Adverse Events and Adverse Device Effects 14
3.3 Risks 14
3.4 Risk to Benefit Rationale 14
3.5 Residual Risks Associated with the Device 14
3.6 Steps to Control or Mitigate Risks 14
4. Study Design 15
4.1 Purpose 15
4.2 Study Design and Scope 15
4.2.1 Subject population 15
4.2.2 Number of study subjects 15
4.2.3 Enrollment period and study duration 15
4.3 Objectives 15
4.3.1 Primary objectives 15
4.3.2 Secondary objectives 16
4.4 Inclusion and Exclusion Criteria 16
4.4.1 Inclusion criteria 16
4.4.2 Exclusion criteria 16
4.5 Informed Consent Process 17
5. Device 17
5.1 Device Description 17
5.2 Device Accountability 18
6. Study Procedures 19
6.1 Screening/Enrollment/Baseline 21
6.2 Implant/Procedure/Post-Procedure 22
6.3 Scheduled Follow-ups 23
6.4 Interim/Unscheduled Visits 23
6.5 Sponsor Representatives 23
6.6 Subject Study Completion 24
6.7 Compromising Factors 24
6.8 Subject Withdrawal 24
7. Compliance Statements 24
8. Serious Adverse Events, Serious Adverse Device Effects, Deaths and Device Deficiencies 25
8.1 Serious Adverse Events 25
8.1.1 Serious adverse device effect 29

8.1.2 Assessing, recording and reporting serious adverse events, serious adverse device effects, and device deficiencies/complaints 29
8.1.3 Subject death 30
8.2 Device Deficiency/Complaint Handling 30
9. Data Management 31
9.1 Data Management Plan 31
9.2 Document and Data Control 31
9.2.1 Document and data traceability 31
9.2.2 Recording data 31
10. Monitoring 32
11. Regulatory Inspections 32
12. Statistical Methods 32
13. Advisory Committees 33
13.1 Steering Committee 33
13.2 Clinical Events Committee 34
13.3 Echocardiography Core Laboratory 34
14. Document Retention 34
15. Amendments to the Clinical Protocol 34
16. Outsourcing of Sponsor Duties and Functions 34
17. Investigation Suspension or Termination 35
17.1 Premature Termination 35
17.2 Study Conclusion 35
18. Publication Policy 36
19. Bibliography 36
Appendix A: Abbreviations 40
Appendix B: Protocol Revision History 41
Appendix C: Declaration of Helsinki 42
Appendix D: Investigator’s Brochure (IB) 43
Appendix E: Echocardiography Core Laboratory Protocols 44
Appendix F: List of Clinical Investigation Sites and Ethics Committees 48
Appendix G: Sample Informed Consent 49
Appendix H: Case Report Forms 61
Appendix I: Device Deficiency/Complaint Handling 62
Appendix J: Modified Rankin Scale (mRS) 64
Appendix K: Intracardiac Echocardiography (ICE) Sub-Study Requirements 65
Appendix L: Cardiovascular Mortality Definition 66

Figures

Figure 1: The Amulet device and key components 18
Figure 2: Study flowchart for subject screening, consent, enrollment and follow-up 19

Tables

Table 1: Model numbers and key dimensions of the Amulet device and delivery system 18
Table 2: Study visits and activities 20
Table 3: Study visits and electronic case report forms (Apppendix H) 20

1. SYNOPSIS

Title of Clinical Study	AMPLATZER™ Amulet™ Observational Post-Market Study
Clinical Study Number	SJM-CIP-10053
Conformité Européenne (CE) Mark	AMPLATZER™ Amulet™: 03 January 2013<br>Change in AMPLATZER™ Amulet™ delivery cable: 23 September 2014
Device	The AMPLATZER™ Amulet™ Left Atrial Appendage Occluder (hereinafter referred to as the "Amulet device") is a transcatheter, self-expanding device constructed from a nitinol mesh and consists of a lobe and a disc connected by a central waist. Polyester patches sewn into both the lobe and disc facilitate occlusion. The lobe has stabilizing wires to improve device placement and retention. The device has threaded screw attachments at each end for connection to the delivery and loading cables. The device has radiopaque markers at each end and at the stabilizing wires that permit visibility during fluoroscopy.<br><br><u>Amulet Device Models</u><br>100099039 9-ACP2-007-016 Pkgd Sterile OUS<br>100099040 9-ACP2-007-018 Pkgd Sterile OUS<br>100099041 9-ACP2-007-020 Pkgd Sterile OUS<br>100099042 9-ACP2-007-022 Pkgd Sterile OUS<br>100099043 9-ACP2-010-025 Pkgd Sterile OUS<br>100099045 9-ACP2-010-028 Pkgd Sterile OUS<br>100099046 9-ACP2-010-031 Pkgd Sterile OUS<br>100099047 9-ACP2-010-034 Pkgd Sterile OUS
Clinical Registry Purpose	To compile real world outcome data on the use of the Amulet device in non-valvular atrial fibrillation (NVAF) subjects
Trial Type	Post-market observational study
Clinical Design	Prospective, multicenter, observational, non-randomized study
Primary Objectives	The Amulet observational post-market study has four primary objectives:<br>1. Assessment of acute (0 - 7 days post procedure) serious adverse events<br>2. Assessment of late (> 7 days post-procedure) serious adverse events through 2 years<br>3. Report the percentage of stroke (ischemic), systemic embolism and cardiovascular death through 2 years<br>4. Report the percentage of bleeding events through 2 years
Secondary Objectives	The Amulet observational post-market study has three secondary objectives:<br>1. Technical success - successful implantation of the Amulet device in the left atrial appendage (LAA)<br>2. Procedural success – technical success and the absence of major adverse

	events during hospitalization. Major adverse events include death, stroke, embolism, pericardial or other major bleeding requiring intervention, device embolization, and major vascular complications<br>3. Report percentage of subjects taking oral anticoagulation (OAC) and antiplatelet drugs through 2 years
Investigators	Electrophysiologist (EP) and/or Interventional Cardiologist (IC)
Clinical Sites	Up to 75 sites in Europe, Middle East and Africa (EMEA), Australia-New Zealand (ANZ), Latin America, Asia-Pacific (AP)<br>*Note: Amulet device regulatory/market approval in each country is required*
Sample Size	1000 subjects
Population	Subjects with NVAF
Inclusion Criteria	1. Subject with history of paroxysmal, persistent or permanent NVAF<br>2. Subject who is 18 years or older, or of legal age to give informed consent specific to state and national law<br>3. Subject who is eligible for an Amulet LAA Occluder device according to current international guidelines and per physician discretion<br>4. Subject who is willing and capable of providing informed consent, participating in all associated study activities
Exclusion Criteria	1. Subject with evidence of an intracardiac thrombus<br>2. Subject with active infection or active endocarditis or other infections producing bacteremia<br>3. Subject where the placement of the device would interfere with any intracardiac or intravascular structures<br>4. Subject with any medical disorder that would interfere with completion or evaluation of clinical study results<br>5. Women of childbearing potential who are, or plan to become, pregnant during the time of the study (method of assessment per physician discretion)<br>6. Subject with LAA anatomy that does not accommodate a device per the sizing guidelines
Post Procedure Care	▪ Monitor the subject until discharge<br>▪ Perform a transthoracic echocardiogram (TTE)/follow up before discharge to confirm the device is positioned correctly and no pericardial effusion is present<br>▪ Follow the instructions for use (IFU) for the drug regimen (dual antiplatelet) post implant

Clinical Follow-up Visits/Testing	Subjects will be followed through two years post implant.<br>**<u>Required Testing</u>**<br>▪ **Baseline evaluation**: Informed consent, history and physical, pregnancy testing for women of childbearing age, 12-lead electrocardiogram (ECG), CHA2DS2-VASc score, HAS-BLED score, Modified Rankin Scale (mRS), medication assessment<br>▪ **Procedure**: Transoesophageal echocardiogram (TOE) or intracardiac echocardiogram (ICE) only when combined with baseline/pre-procedural TOE, angiogram, adverse event assessment<br>▪ **Post procedure/Pre-Discharge**: TTE, medication assessment, adverse event assessment<br>▪ **1M to 3M**: TOE, medication assessment, adverse event assessment<br>▪ **6M (+/- 2 weeks):** Phone contact follow-up for medication and AE assessment<br>▪ **12M (+/- 6 weeks):** Medication and adverse event assessment<br>▪ **24M (+/- 12 weeks):** Phone contact follow-up for medication and adverse event assessment<br>▪ **Interim/unscheduled visits**: per physician discretion
Suspected Stroke/TIA Evaluation	▪ All subjects suspected of a stroke should be seen by a stroke/TIA neurologist for evaluation and appropriate neurological testing done (i.e. magnetic resonance imaging (MRI), computed tomography (CT)<br>▪ Modified Rankin Scale (**Appendix J**)<br>▪ TOE should be conducted to verify the presence of device-related thrombus
Clinical Procedures	▪ Angiography with fluoroscopy<br>▪ TOE with color flow Doppler<br>▪ TTE post-procedure/pre-discharge<br>▪ 12-lead ECG
Clinical Duration	The total study duration is expected to be 4 years. This observational study will begin March 2015 with the first subject enrolled first half of 2015 and the last subject enrolled second half of 2016, the 2 year follow-up will be complete second half of 2018
St. Jude Medical	St. Jude Medical, Inc.<br>Global Clinical Affairs Organization<br>5050 Nathan Lane North<br>Plymouth, MN 55442<br>USA<br>Main +1 6517565400

1.1 Study Contacts

Clinical Department, LAA Program
St. Jude Medical, Inc.
Global Clinical Affairs Organization
5050 Nathan Lane North
Plymouth, MN 55442
USA
Main +1 6517565400
Fax +1 8772572381

Sarah Gallagher
Clinical Programs Manager
LAA Occlusion
St. Jude Medical
5050 Nathan Lane North
Plymouth, MN 55442
USA
Direct +1 6517566937
Main +1 7635139227
Mobile +1 6514855744
Fax +1 8772572381
sgallagher02@sjm.com

Anne Marie Harcarik
Sr. Director Clinical Affairs
LAA Occlusion
St. Jude Medical
5050 Nathan Lane North
Plymouth, MN 55442
USA
Direct +1 6517566469
Main +1 7635139227
Mobile +1 6122195601
Fax +1 8772572381
AHarcarik@sjm.com

Hindrik Robbe
Vice President Field Clinical Affairs EMEA
St. Jude Medical
The Corporate Village | Da Vincilaan 11 Box F1
1935 Zaventem Vlaams Brabant
Belgium
Main +32 27746849
Mobile +32 499544060
HRobbe@sjm.com

Catherine Bourgeois
Vice President Field Clinical Affairs AP & Japan
St. Jude Medical
17 Orion Road
Lane Cove NSW 2066
Australia
Main +61 299361235
Mobile +61 410330259
CBourgeois@sjm.com

1.2 Certificate History

Date	Action
03 January 2013	Amulet CE Mark
23 September 2014	Change in Amulet delivery cable

2. BACKGROUND AND JUSTIFICATION

The loss of mechanical efficiency during atrial fibrillation (AF)* leads to insufficient contraction in the left atrium (LA)[1]. Stagnation of blood flow in the LA leads to hypercoagulability and thus increased risk for thrombus formation in the LA or LAA. Approximately 90% of all thrombi in subjects with NVAF forming in the LA originate in the LAA[2]. The thrombus formation, in turn, exposes the subject to thromboembolic events. Approximately 6% of embolic events result in peripheral embolism as opposed to ischemic stroke[3].

Echocardiographic risk factors for LAA thrombus formation include echocardiographic evidence of decreased LAA flow velocity and spontaneous echo contrast within the LA and LAA[4, 5]. The normal flow pattern of the LAA is the ejection of blood from the appendage following atrial contraction at a velocity greater than 40cm/s[6]. Agmon et al. found that the relative risk reduction of ischemic stroke was 2.6 times greater in subjects with LAA flow velocities < 20 cm/s than those with higher LAA velocities[7].

Ischemic strokes in subjects with AF caused major deficits as compared to ischemic strokes in subjects with carotid disease. Subjects with AF had silent infarcts in the SPINAF study in which a CT scan was performed both initially and then again at the end of the average follow-up period of 1.7 years[8].

Non-valvular AF subjects were assessed to determine the risk of stroke based on the existence of independent risk factors. In a study by Gage et al., the $CHADS_2$ index was found to be the most accurate in predicting the risk of stroke in subjects with AF[9]. The $CHADS_2$ score assigns one point each for the presence of congestive heart failure, hypertension, age greater than 75, and diabetes mellitus and two points for history of stroke or transient ischemic attack (TIA). The study found that AF subjects who were not treated with antithrombotics had an increased risk of stroke from 1.5% to 18.2% based on scores of one and six respectively.

A study by Go et al., reviewed outcome data (11,526 subjects) in a large primary care setting and confirmed that thromboembolic risk increases progressively with $CHADS_2$ score[3]. The study also noted that oral anticoagulation with warfarin reduces the risk of stroke in most subjects with the exception of those at lowest risk ($CHADS_2$ score of zero) and highest risk ($CHADS_2 \geq 5$) for stroke.

In a meta-analysis conducted by Andersen et al., warfarin was found to be superior to aspirin and placebo in reducing the risk of both stroke and systemic embolism in subjects with NVAF[10]. Hart et al. reported that adjusted dose warfarin reduces stroke by 64% (6 trials) and antiplatelet agents reduce stroke risk by 22%[11]. The study also reported that risk of intracranial hemorrhage was doubled with adjusted dose warfarin compared with aspirin, although the absolute risk increase was by adjusted-dose warfarin vs. control.

Subjects are often poorly compliant with anticoagulation, and anticoagulants are contraindicated in selected subsets of subjects. The reasons given for being contraindicated included prior bleed, patient refusal/preference, high bleeding risk, frequent falls/frailty, need for dual antiplatelet therapy, unable to adhere/monitor warfarin, comorbid illness, prior intracranial hemorrhage, allergy, occupational risk and pregnancy.

See **Appendix A for a listing of abbreviations used throughout this document*

Recently, new drugs have been developed with less dietary and pharmacological interactions and less stringent requirements for frequent INR monitoring. Major trials such as RE-LY and ROCKET AF demonstrated that dabigatran and rivaroxiban are non-inferior to warfarin in the prevention of stroke and systemic embolism[12, 13]. The ARISTOTLE Trial demonstrated apixaban was superior to warfarin in preventing stroke or systemic embolism, caused less bleeding, and resulted in lower mortality in subjects with atrial fibrillation[14]. The ENGAGE AF-TIMI 48 Trial demonstrated both once-daily regimens of edoxaban were noninferior to warfarin with respect to the prevention of stroke or systemic embolism and were associated with significantly lower rates of bleeding and death from cardiovascular causes[15].

Left atrial appendage occlusion (LAAO) is considered a viable alternative to OAC therapy for stroke prevention in patients with NVAF[16-26]. Evidence supporting LAAO mainly consists of PROTECT AF and PREVAIL, the major randomized studies published to date[16-19]. Five-year results of PROTECT AF showed superiority of the Watchman device in mortality and stroke reduction compared to optimal medical treatment with warfarin[19]. The Watchman device is constructed of nitinol (a nickel/titanium alloy) and is composed of 10 fixation anchors around the device perimeter that are designed to secure the device in the LAA (Boston Scientific, Minneapolis, MN, USA).

There are several other endocardial LAAO devices currently in pre-clinical/clinical trial testing phases and one of these (WaveCrest) has CE Mark approval[27]:

- Occlutech LAA Occluder (Occlutech, Helsingborg, Sweden)
- Lifetech LAmbre (Lifetech, Shenzhen, PRC)
- Sideris Transcatheter Patch (Custom Medical Devices, Texas, USA)
- Ultrasept (Cardia, Eagan, MN, USA)
- Pfm ((Pfm Medical, Inc., Carlsbad, CA, USA)
- WaveCrest LAAO (Coherex Medical, Salt Lake City, UT, USA)

AMP LAT ZER™ Cardiac Plug and the AMP LA T ZER™ Amulet™ device

Both the AMPLATZER Cardiac Plug (ACP) and AMPLATZER Amulet devices have CE Mark approval. The ACP device (St Jude Medical, St. Paul, MN, USA) is a self-expandable nitinol device, with fixation anchors. The ACP device is a first generation device based on AMPLATZER occluder technology specifically designed for LAAO. Refer to the Investigator's Brochure (**Appendix D**) for data published on the ACP device.

The ACP device demonstrated favorable feasibility and safety results in observational studies in Europe[28-30]. Professor Park's article details an investigator–initiated retrospective data collection to evaluate and demonstrate the initial European experience in patients treated with the ACP device between December 2008 and November 2009. Favorable ACP registry study results were presented at EURO PCR in 2012 and 2014[29-30].

The Amulet device is a second-generation ACP device. To date there have been two single center self-reported studies of initial use of the Amulet device. The first reported use by Lam et al. involved a retrospective review of 17 subjects from implant to 90 days follow-up at the Cardiovascular Center in Frankfurt[31]. The second reported use by Freixa et al. involved 25 subjects enrolled at Barcelona University and patients were followed for 3 months[32]. Both of these single center studies report high

implant success rates however, long-term safety and efficacy analysis were not conducted due to the short follow up period.

Results from a multicenter study involving 22 sites, 1,047 consecutive patients and the ACP device showed high procedural success and a favorable outcome for the prevention of AF-related thromboembolism[33].

The aim of this observational post-market study is to compile real world outcome data on the use of the CE marked Amulet device in NVAF subjects.

3. RISKS AND BENEFITS

3.1 Anticipated Clinical Benefits

For subjects who can take OAC medication the potential benefit is that subjects may not need to be on long-term warfarin, or other OAC therapy and thus will not be exposed to associated complications. This device may also provide potential benefits to subjects who cannot take OAC. Subjects who receive the device may also experience a decrease in the associated risk of thromboembolism.

3.2 Anticipated Adverse Events and Adverse Device Effects

Anticipated adverse events and adverse device effects may occur during and after the Amulet device implant procedure. Serious adverse event definitions are provided in Section 8.

3.3 Risks

The risks associated with the Amulet device implant procedure are similar to those of other cardiac catheterization procedures. Radiation risks associated with the procedure are comparable to a diagnostic catheterization procedure.

The risks associated with the implanted Amulet device are similar to other implantable cardiac LAAO devices. Refer to Section 8 for a complete list of potential anticipated serious adverse events, which are also included in the IFU included in the Investigator's Brochure (**Appendix D**).

3.4 Risk to Benefit Rationale

Percutaneous LAAO offers an alternative to physicians who are caring for patients with AF who are at high risk for both bleeding and stroke.

3.5 Residual Risks Associated with the Device

The Amulet device is market approved in all the countries where the study will be conducted. The risk profile of the Amulet device implant procedure is described in the labeling for the CE marked device, the risk analysis report, the IFU (included in the IB in **Appendix D**), and Section 8 of this protocol. The overall events from a risk perspective are similar to the risks with minimally invasive implantable therapies. Complication rates are within expected ranges and the risk/benefit profile is similar to alternate therapies.

3.6 Steps to Control or Mitigate Risks

The Sponsor will select investigators qualified by training and experience to participate in this study. Participating sites will be selected based upon qualifications of the primary investigator. During the study, sites may be subject to quality assurance audits by the Sponsor (or designee), as

well as monitoring visits to assess data integrity and study compliance. Additionally, the study will have an appointed independent Clinical Event Committee (CEC) that will review and adjudicate all reported serious adverse events. Any additional risk is mitigated with vigilance reporting for a CE marked product.

4. STUDY DESIGN

4.1 Purpose

The Amulet device is a transcatheter, self-expanding nitinol device intended for use in preventing thrombus embolization from the LAA. The purpose of the Amulet observational post-market study is to compile real world outcome data on the use of the CE marked Amulet device in NVAF subjects.

4.2 Study Design and Scope

The Amulet device will be clinically evaluated through this observational post-market study. The study design is a prospective, multicenter, non-randomized observational post-market study, where all subjects eligible for enrollment will receive the Amulet device.

4.2.1 Subject population

The subject population will include participants that are $\geq$ 18 years old and diagnosed with NVAF.

4.2.2 Number of study subjects

Approximately 1000 subjects will be enrolled in the study at up to 75 sites in EMEA, ANZ, Latin America, and AP. A list of clinical investigation sites and ECs will be kept under separate cover and is available upon request (**Appendix F**).

4.2.3 Enrollment period and study duration

Enrollment in the study will be complete within approximately 24 months of the first subject enrollment. As the study requires follow-up visits through at least 2 years, the total study duration is expected to be 4 years.

4.3 Objectives

4.3.1 Primary objectives

The Amulet observational post-market study has four primary objectives:

1. Assessment of acute (0 - 7 days post procedure) serious adverse events
2. Assessment of late (> 7 days post-procedure) serious adverse events through 2 years
3. Report the percentage of stroke (ischemic), systemic embolism, and cardiovascular death through 2 years
    - Cardiovascular death is defined as (see **Appendix L**) death due to proximate cardiac cause, death caused by non-coronary, non CNS vascular conditions, death from vascular CNS causes, hemorrhagic stroke, ischemic stroke, all procedure related deaths, sudden or unwitnessed death and death of an unknown cause[34]

4. Report the percentage of bleeding events through 2 years

4.3.2 Secondary objectives

The Amulet observational post-market study has three secondary objectives:

1. Technical success – successful implantation of the Amulet device in the LAA
2. Procedural Success – technical success and the absence of major adverse events during hospitalization. Major adverse events include death, stroke, embolism, pericardial or other major bleeding requiring intervention, device embolization, and major vascular complications
3. Report percentage of subjects taking OAC and antiplatelet drugs through 2 years

4.4 Inclusion and Exclusion Criteria

Subjects, who meet all of the inclusion criteria and none of the exclusion criteria, are eligible to participate in this study.

All subjects who sign the informed consent will be assigned a study identification number and this will be recorded on an Inclusion/Exclusion electronic case report form (eCRF). The subject ID number will be unique to each individual and will allow the participant to be linked if necessary, to name, alternative identification or contact information. To ensure subject privacy and confidentiality of data, subject ID numbers will be used and maintained by study site personnel for the duration of the study.

4.4.1 Inclusion criteria

To participate in this clinical study, the subject must meet all of the following four inclusion criteria:

1. Subject with a history of paroxysmal, persistent or permanent NVAF
2. Subject who is 18 years or older, or of legal age to give informed consent specific to state and national law
3. Subject who is eligible for an Amulet LAAO device according to current international guidelines and per physician discretion
4. Subject who is willing and capable of providing informed consent, participating in all testing associated with this clinical study

4.4.2 Exclusion criteria

Subjects are not eligible for clinical study participation if they meet any of the following six exclusion criteria:

1. Subject with evidence of an intracardiac thrombus
2. Subject with active infection or active endocarditis or other infections producing bacteremia
3. Subject where the placement of the device would interfere with any intracardiac or intravascular structures

4. Subject with any medical disorder that would interfere with completion or evaluation of clinical study results
5. Women of childbearing potential who are, or plan to become, pregnant during the time of the study (method of assessment per physician discretion)
6. Subject with LAA anatomy that does not accommodate a device per the sizing guidelines

4.5 Informed Consent Process

The process for obtaining informed consent must comply with the ethical principles defined in the current version of the Declaration of Helsinki (**Appendix C**).

Prior to enrollment in the clinical study, all subjects will be consented, as required by applicable regulations and the site's ethics committee (EC). Informed consent must be obtained from each subject prior to the device implant procedure. The consent form must be signed and dated by the subject and by the person obtaining the consent, where applicable.

The principal investigator (PI) or his/her authorized designee will conduct the informed consent process. This process will include a verbal discussion with the subject on all aspects of the clinical study that are relevant to the subject's decision to participate, and the subject should be allowed adequate time to review and ask questions.

Documentation that informed consent was obtained will be recorded on the eCRF and in the subject's medical record. The signature date on the consent form should be referenced. The original signed informed consent should be filed in the subject's study chart and a copy of the signed consent form given to the subject per EC requirements.

A template of the informed consent form is provided in **Appendix G**. Any changes to the template must be approved by SJM prior to ethics committee review and approval.

A subject cannot be consented to the study prior to EC approval of the informed consent form. The subject shall be provided the informed consent form that is written in a language understandable to the subject and has been approved by the site's EC.

The subject is enrolled in the study when consent has been obtained and the dilator/delivery system is introduced. Failure to obtain informed consent from a subject prior to study enrollment must be reported to SJM as soon as possible and to the reviewing site's EC consistent with the site's EC reporting requirements.

5. DEVICE

5.1 Device Description

The Amulet device (**Figure 1**) is constructed from a nitinol mesh and consists of a lobe and a disc connected by a central waist. The lobe ranges in diameter from 16 to 34 mm (**Table 1**) and has stabilizing wires for device placement and retention. The disc is larger in diameter than the lobe, ranging from 20 to 41 mm; both the disc and the lobe contain polyester fabric to facilitate LAAO. There are threaded screw attachments at either end of the device for connection to the delivery and loading cable. Radiopaque markers at either end of the device and at the location of the stabilizing wires allow for predictable placement of the device. The stabilizing wires and polyester patch are

secured to the device using polyester thread. A platinum/iridium thread is attached to the nitinol braid.

Accessories packaged with the Amulet device include the loader, loading cable, loading cable vise, delivery cable, delivery cable vise, and hemostasis valve. The Amulet device is recommended for delivery using the AMPLATZER TorqVue 45 x45 Delivery Sheath (12F and 14F). Refer to the Investigator's Brochure (IB) (**Appendix D**) which includes the IFU for additional information.

Figure 1: The Amulet device and key components

A. Screw attachments
B. Waist of device
C. Lobe
D. Stabilizing wires
E. Disc

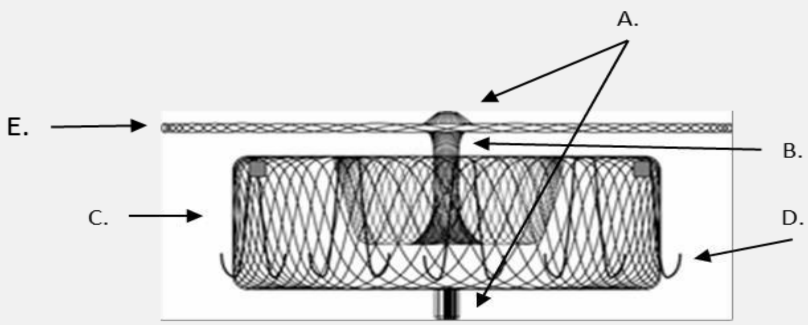

Table 1: Model numbers and key dimensions of the Amulet device and delivery system

Part Number	Amulet Device Size	Left Atrial Disc Size (mm)	Lobe Length (mm)	# of stabilizing wires	TorqVue Delivery System
9-ACP2-007-016	16mm	22	7.5	6	12F*
9-ACP2-007-018	18mm	24	7.5	6	12F*
9-ACP2-007-020	20mm	26	7.5	8	12F*
9-ACP2-007-022	22mm	28	7.5	8	12F*
9-ACP2-010-025	25mm	32	10	8	12F*
9-ACP2-010-028	28mm	35	10	10	14F
9-ACP2-010-031	31mm	38	10	10	14F
9-ACP2-010-034	34mm	41	10	10	14F

**Per the IFU, if a 14 Fr delivery sheath is selected for use with a 16 mm-25mm device, tightly connect the 14 Fr sheath adaptor to the delivery sheath hub*

5.2 Device Accountability

The Amulet device has CE Mark approval and is market released in the countries where this study will take place. Therefore, there are no additional tracking requirements for this study. Information regarding opened, introduced, and implanted devices will be recorded on the Procedure eCRF.

6. STUDY PROCEDURES

This study will be conducted in accordance with the clinical protocol and IFU included in the IB (**Appendix D**). All persons participating in the conduct of the study will be qualified by education, training, or experience to perform study-related tasks.

The study will not commence until SJM receives written approval from the EC and relevant regulatory authorities, and all required documents have been collected from the participating sites.

Figure 2 below describes the study flowchart for subject screening, consent, enrollment and follow-up through the 2-year follow-up visit. **Table 2** below outlines the testing and assessments required per study visit interval. **Table 3** below outlines the eCRFs to be completed for enrolled subjects that receive the device, and as per the recommended study visit intervals.

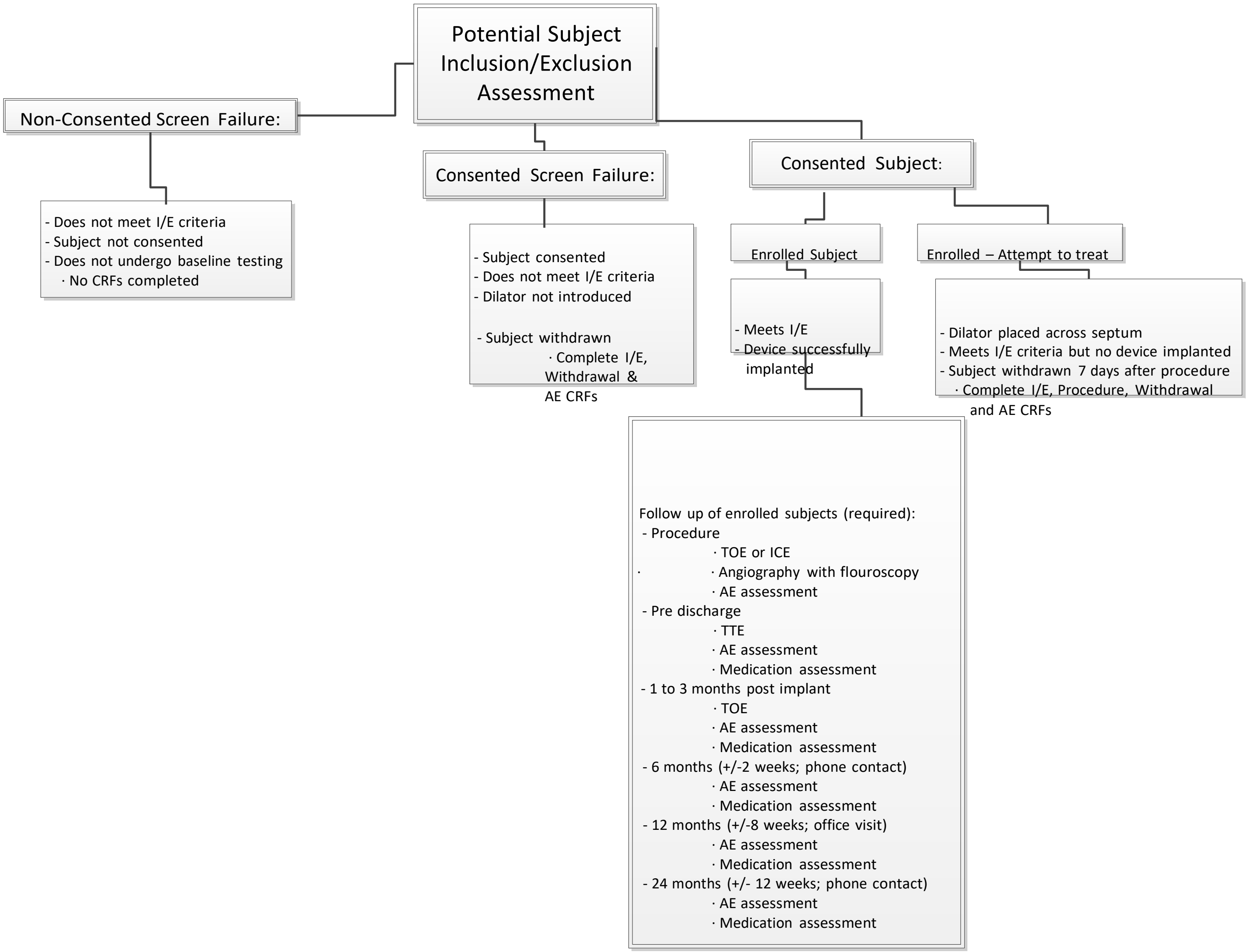

Figure 2: Study flowchart for subject screening, consent, enrollment and follow-up

Table 2: Study visits and activities

Visit / Study Activity	Enrollment & Baseline	Procedure	Discharge	1-3M	6M Phone Visit (+/- 2 weeks)	12M (+/- 6 weeks)	24M Phone Visit (+/- 12 weeks)	Interim Follow-up
Informed Consent Process	X							
- History and physical<br>- Cardiovascular and medical exam<br>- CHA_2DS_2-VASc score<br>- HAS-BLED score	X							
12-lead ECG	X							
Pregnancy Test[1]	X							
Medication Assessment	X		X	X	X	X	X	X
Modified Rankin Scale[2]	X							X[4]
Angiography		X						
TTE			X					
TOE	X[,5]	X[3,5]		X				X[4]
ICE		X[6]						
Adverse Event Assessment		X	X	X	X	X	X	X

[1]Pregnancy test for women of childbearing potential
[2]Perform additional Modified Rankin Scale assessment 90 days after a confirmed stroke or TIA
[3]CT may be used as additional information and performed complimentary to the TOE, per institutional standard of care
[4]Complete if stroke or TIA is confirmed
[5]The Baseline TOE is optional if a TOE is performed on the day of procedure to rule out the presence of intracardiac thrombus (including left atrial appendage thrombus) and to assess the size and shape of the LAA
*[6]ICE may be performed for placement of the device in a sub-set of centers pre-approved by SJM (see **Appendix K**)*

Table 3: Study visits and electronic case report forms (Appendix H)

Visit / eCRF	Enrollment & Baseline	Procedure	Discharge	1-3M	6M Phone Visit	12M	24M Phone Visit	Interim Follow-up
Baseline	X							
Inclusion/ Exclusion	X							
Procedure		X						
Follow Up			X	X	X	X	X	X*
Medication	X	X	X	X	X	X	X	X
Protocol Deviation	X**	X	X	X	X	X	X	X

**Complete if stroke or TIA is confirmed*
*** Complete if informed consent/informed consent form was not appropriately obtained/completed, visit out of window, test not completed.*

Additional eCRFs, reports and assessment noted below should be completed upon occurrence, and/or as applicable:

- Withdrawal eCRF
- Adverse Event eCRF
- Protocol Deviation eCRF
- Death eCRF
- Follow-up eCRF for interim/unscheduled visits
- Modified Rankin Scale **(Appendix J**) (complete 90 days after confirmed stroke /TIA)

If a suspected stroke/TIA has occurred, the subject should be seen by a stroke/TIA neurologist for evaluation and appropriate neurological testing (i.e. CT or MRI, and CTA or MRA). If a stroke or TIA is confirmed, a TOE is required as soon as possible to confirm device placement, LAA flow parameters and presence/absence of device-related thrombus. See Section 8.1 for further details.

6.1 Screening/Enrollment/Baseline

The following baseline and enrollment activities are performed as part of the screening process:

- Informed consent process
- History and physical
- Cardiovascular and medical exam
- Pregnancy testing for women of childbearing age
- 12-lead ECG
- TOE (optional if a TOE is performed on the day of procedure)
- Modified Rankin Scale
- Medication assessment
- CHA2DS2-VASc score:

**CHA_2DS_2-VASc**	**Score**
**C**ongestive heart failure/LV dysfunction	1
**H**ypertension	1
**A**ge $\geq$ 75	2
**D**iabetes mellitus	1
**Stroke/TIA/TE**	2
**V**ascular disease [prior myocardial infarction, peripheral artery disease or aortic plaque]	1
**A**ge 65-74	1
**S**ex category [i.e. female gender]	1

- HAS-BLED score:

HAS-BLED	Score
**H**ypertension history (uncontrolled, > 160 mmHg systolic)	1
**A**bnormal renal function/renal disease (dialysis transplant, creatinine > 2.6 mg/dL or > 200µmol/L)	1
Liver disease (cirrhosis, bilirubin > 2x normal AST/ALT/AP > 3x normal)	1
**S**troke history	1
**B**leeding, anemia, or predisposition to bleeding	1
**L**abile INR (Unstable/high INR’s, or poor time (< 60% time in therapeutic range)	1
**E**lderly - Age ≥ 65 years	1
**D**rugs - Medication usage predisposing to bleeding (antiplatelet agents, NSAIDs)	1
Alcohol usage history (> 8 drinks/week)	1

The principal investigator or delegated study personnel is responsible for screening potential subjects to determine subject eligibility for the study. Enrollment information (date of consent, inclusion/exclusion information, etc.) will be recorded in the subject’s hospital records and on the Baseline and Inclusion/Exclusion eCRF. The Inclusion/Exclusion eCRF should be submitted within one week and will serve as notification of enrollment to the Sponsor upon receipt.

If a subject does not meet all inclusion criteria or meets any of the exclusion criteria, the subject cannot participate in the study. The Baseline/Screening and Inclusion/Exclusion eCRFs should be completed, in addition to the Withdrawal eCRF (consented screen failure), as applicable. If the subject was enrolled (i.e. consented and the dilator/delivery system introduced), but meets the exclusion criteria during the procedure, the Baseline, Inclusion /Exclusion, Procedure, and Withdrawal eCRFs (complete 7 days after the attempted procedure) should be completed.

Serious adverse events will be reported from the time the dilator/device delivery system has been introduced to the time the subject concludes the study. An Adverse Event eCRF should be completed, as applicable.

6.2 Implant/Procedure/Post-Procedure

After the subject has met preliminary inclusion criteria, the implant procedure can be scheduled.

The baseline TOE is optional if a TOE is performed on the day of procedure to rule out the presence of intracardiac thrombus (including left atrial appendage thrombus) and to assess size and shape of the LAA.

See the IFU for procedural and post-procedural instructions.

The post-procedure care should involve the following (refer also to **Table 2**):

- Perform a TTE prior to discharge to ensure the device is positioned correctly and no pericardial effusion is present
- Aspirin (or an alternate antiplatelet) is recommended for subjects 6 months post implant
- Clopidogrel (or an alternate antiplatelet) is recommended for implanted subjects and prescription should follow standard of care
- Appropriate endocarditis prophylaxis is recommended
- Instruct the subject on when to seek medical attention
- Provide routine standard of care follow-up, including echocardiography, for evaluation of residual shunt (flow) and adverse events (including thrombus formation)
- Go to www.amplatzer.net/device-registration-international.html to print the temporary subject identification card. Complete this card and provide it to the subject

6.3 Scheduled Follow-ups

Required follow-up visits will be at 1-3 months, 6-months telephone follow-up, 12-months, and 24-month telephone follow-up. Refer to **Table 2** for required follow-up testing and assessments.

6.4 Interim/Unscheduled Visits

Interim/unscheduled visits will be considered those that occur in addition to the required visit interval schedule. If an unscheduled or interim visit occurs, a Follow-up eCRF should be completed to capture data collected at the visit as well as pertinent additional eCRFs (medication and adverse event assessments as applicable). In the event of a confirmed stroke or TIA, the Modified Rankin Scale eCRF should be completed 90 days after the event and echo imaging should be submitted to the core lab.

6.5 Sponsor Representatives

Trained Sponsor personnel may perform certain study activities to ensure compliance to the clinical protocol and may provide technical expertise. Monitoring may be performed by SJM and/or authorized designees according to the International Organization for Standardization (ISO) 14155 and MEDDEV guidelines for post market studies; and applicable SJM standard operating procedures and work instructions. Qualified monitors will ensure investigators comply with this clinical protocol and ISO 14155 and MEDDEV post market requirements.

To ensure study personnel accept, understand and complete their assigned responsibilities, monitors, field clinical personnel, and/or clinical country managers, may perform periodic site visits during the course of the study. These actions will help to ensure the continued acceptability of the facilities, compliance to the clinical protocol and relevant regulations, and the maintenance of complete records.

Monitoring will include review and resolution of missing or inconsistent results and source document verification (i.e. comparison of submitted study results to original reports) to assure the accuracy of the reported data.

Sponsor representatives will periodically request source documents, resolution of discrepancies, submission of echocardiography imaging etc. for data cleaning and reporting purposes.

6.6 Subject Study Completion

After the 2-year follow-up visit is completed, subjects will be discontinued from the study and return to receiving medical care per their physician's recommendations.

6.7 Compromising Factors

This is an observational post-market study evaluating real-world outcomes in subjects with LAAO. There are no anticipated or foreseeable factors that may compromise the outcome of the study or interpretation of results.

6.8 Subject Withdrawal

Subjects must be informed about their right to withdraw from the study at any time and for any reason without sanction, penalty, or loss of benefits to which the subject is otherwise entitled. Withdrawal from the study will not jeopardize their future medical care or relationship with the investigator. Subjects will be asked to specify the reason for their termination, but have the right not to answer.

The investigator may decide to withdraw a subject from the study at any time with reasonable rationale. The subject's future care will not be influenced by a decision, voluntary or otherwise, to withdraw from the study. All reasonable efforts should be made to retain the subject in the study until completion of the study.

Reasons for subject withdrawal include, but are not limited to:

- Subject withdraws consent
- Subject refuses to continue their participation
- Subject does not meet the inclusion/exclusion criteria (withdraw attempt to treat subjects 7 days after the date of the attempted procedure)
- Subject is deceased (event or symptoms pertaining to outcome of death must be documented on an Adverse Event eCRF)
- Subject's participation is terminated by the PI or investigator, although the subject consented, since participation is no longer medically appropriate
- Subject is 'lost to follow up' (subject does not adhere to the scheduled follow up visits but has not explicitly requested to be withdrawn from the study)
- Site personnel should make reasonable efforts to locate and communicate with the subject in order to achieve subject compliance to the scheduled follow up visits

If a subject withdraws from the study, the site will record the subject's reasons for withdrawal on the Withdrawal eCRF.

7. COMPLIANCE STATEMENTS

The study will be performed in accordance with the most current versions of the World Medical Association (WMA) Declaration of Helsinki (**Appendix C**), ISO 14155, MEDDEV post market requirements and any regional and/or national regulations and will be compliant to these International Standards and any regional and national regulations, as appropriate.

The investigator will not enroll subjects or obtain informed consent from any subject prior to obtaining EC approval, if applicable, and authorization from the Sponsor in writing for the study. The subject must sign the study informed consent form prior to any study-related procedures.

If additional requirements are imposed by the EC, those requirements will be followed, if appropriate. If any action is taken by an EC, and regulatory requirements with respect to the study, that information will be forwarded to SJM.

As the Sponsor, SJM has taken general liability insurance in accordance with the requirements of applicable local laws. Appropriate SJM country representatives will be utilized to interpret the requirements regarding the type of insurance that will be provided to subjects, and such information will be incorporated into the informed consent form, as applicable. If required, additional subject coverage or study specific insurance may also be provided by the Sponsor.

8. SERIOUS ADVERSE EVENTS, SERIOUS ADVERSE DEVICE EFFECTS, DEATHS AND DEVICE DEFICIENCIES

Risks associated with the Amulet device are similar to other implantable cardiac occlusion devices. Some of the potential risks include but are not limited to those listed below. The definitions noted below are used for the purposes of this study. Radiation risks associated with the procedure are comparable to a diagnostic catheterization procedure.

Only serious adverse events (SAEs) will be collected for this study. Serious adverse events are classified as serious adverse events or serious adverse device effects (SADEs). A planned hospitalization for a pre-existing condition is not considered a serious adverse event.

8.1 Serious Adverse Events

A serious adverse event is an adverse event that led to:

- Death
- A serious deterioration in the health of the subject, that either resulted in:
  - A life-threatening illness or injury OR
  - A permanent impairment to a body structure or a body function OR
  - An in-patient or prolonged hospitalization OR
  - A medical or surgical intervention to prevent life-threatening illness or injury or permanent impairment to a body structure or a body OR
  - A malignant tumor OR
  - Fetal distress, fetal death or a congenital abnormality or birth defect

Potential serious adverse events include but are not limited to:

- **Air Embolus** – symptomatic event resulting from the introduction of air into the circulatory system
- **Allergic reaction** – idiosyncratic reaction to the device implanted or to nickel
- **Anesthesia reaction** – undesired reaction to anesthetic agent
- **Arrhythmia** – cardiac rhythm disturbance
- **Bleeding** – **see definition of bleeding as defined by BARC at end of section*

- **Cardiac arrest** – failure of the heart to contract
- **Cardiac tamponade** – constriction of the heart causing inefficient contraction resulting from accumulation of excess fluid in the pericardium
- **Death** – permanent cessation of all vital bodily functions
- **Device embolization** – movement of the device from its intended location
- **Device migration** – movement of the device within its intended location
- **Embolic event** – acute vascular insufficiency or occlusion of the extremities or any non-central nervous system organ associated with clinical, imaging, surgical/autopsy evidence of arterial occlusion in the absence of other likely mechanism (e.g., trauma, atherosclerosis, or instrumentation). When there is presence of prior peripheral artery disease, angiographic or surgical or autopsy evidence is required to show abrupt arterial occlusion
- **Fever** – defined as a body temperature > 37.5 or 38.3 °C (99.5 or 100.9 °F)
- **Foreign body embolization** – movement of device material, delivery system material, or other material from its intended location
- **Hypotension** – sustained systolic blood pressure < 90 mmHg
- **Hypertension** – systolic blood pressure of > 160 mmHg
- **Infection** – invasion and growth of a pathogenic organism within the body
- **Multi-Organ Failure –** the failure of two or more systems, such as the cardiovascular and renal systems, and is a common consequence of sepsis (the presence of bacteria in the blood) and of shock (very low blood pressure)
- **Myocardial infarction (heart attack)** – the death of heart muscle from the sudden blockage of a coronary artery by a blood clot
- **Perforation** – physical penetration of a vessel or the myocardium
- **Pericardial effusion** – abnormal fluid collection around the heart without hemodynamic compromise
- **Renal failure/dysfunction** – inability of kidneys to perform normal functions
- **Respiratory failure** – inability of the lungs to perform normal functions
- **Seizure** – uncontrolled electrical activity in the brain, which may produce a physical convulsion, minor physical signs, thought disturbances, or a combination of symptoms
- **Significant Residual Flow –** flow > 3 mm jet into the LAA
- **Stroke** – an acute episode (lasting > 24 hours) of focal or global neurological dysfunction caused by brain, spinal cord, or retinal vascular injury as a result of hemorrhage or infarction. Strokes are characterized as follows[35]:
    - **Ischemic Stroke**: an acute episode of focal cerebral, spinal, or retinal dysfunction caused by infarction of the central nervous system tissue. Hemorrhage may be a consequence of ischemic stroke. In this situation, the stroke is an ischemic stroke with hemorrhagic transformation and not a hemorrhagic stroke.
    - **Hemorrhagic Stroke**: an acute episode of focal or global cerebral or spinal dysfunction caused by intraparenchymal, intraventricular, or subarachnoid hemorrhage

- **Undetermined Stroke**: an acute episode of focal or global neurological dysfunction caused by presumed brain, spinal cord, or retinal vascular injury as a result of hemorrhage or infarction but with insufficient information to allow categorization as ischemic or hemorrhagic stroke

- **Thrombus Formation** – a blood clot
- **Transient Ischemic Attack** (TIA)– a transient episode (lasting ≤ 24 hours) of focal neurological dysfunction caused by brain, spinal cord, or retinal ischemia, *without* acute infarction on brain imaging
- **Valvular regurgitation/insufficiency** – backflow of blood during contraction of the heart; caused by a defective heart valve
- **Vascular access site injury** – damage at vascular access site (e.g., AV fistula, hematoma, and aneurysm)
- **Vessel Trauma/Injury** – traumatic injuries that damage an artery or vein

***Bleeding**
Classified as Type 0 – 5 according to the following BARC definitions[36]:

Type 0: No bleeding

Type 1: Bleeding that is not actionable and does not cause the patient to seek unscheduled performance of studies, hospitalization, or treatment by a healthcare professional; may include episodes leading to self-discontinuation of medical therapy by the patient without consulting a healthcare professional

Type 2 (minor): Any overt, actionable sign of hemorrhage (e.g. more bleeding than would be expected for a clinical circumstance, including bleeding found by imaging alone) that does not fit the criteria for Type 3, 4, or 5 but does meet at least one of the following criteria: (1) requiring nonsurgical, medical intervention by a healthcare professional, (2) leading to hospitalization or increased level of care, or (3) prompting evaluation

Type 3 (major):
Type 3a:
- Any transfusion with overt bleeding
- Overt bleeding plus a hemoglobin drop of ≥ 3 to < 5 g/dL (provided hemoglobin drop is related to bleeding)

Type 3b:
- Overt bleeding plus hemoglobin drop ≥ 5 g/dL (provided hemoglobin drop is related to bleed)
- Cardiac tamponade
- Bleeding requiring surgical intervention for control (excluding dental/nasal/skin/ hemorrhoid)
- Bleeding requiring intravenous vasoactive drugs

Type 3c:

- Intracranial hemorrhage including subdural hemorrhages (does not include microbleeds or hemorrhagic transformation, does include intraspinal)
- Subcategories confirmed by autopsy or imaging or lumbar puncture
- Intraocular bleed compromising vision

Type 4: Coronary artery bypass graft (CABG)–related bleeding:

- Perioperative intracranial bleeding within 48 hours
- Reoperation after closure of sternotomy for the purpose of controlling bleeding
- Transfusion of ≥ 5 units whole blood or packed red blood cells within a 48-hour period (only allogenic transfusions are considered transfusions for CABG-related bleeds)
- Chest tube output ≥ 2 L within a 24-hour period

Notes: If a CABG-related bleed is not adjudicated as at least a type 3 severity event, it will not be classified as a bleeding event. If a bleeding event occurs with a clear temporal relationship to CABG (i.e. within a 48-hour timeframe) but does not meet type 4 severity criteria, it will not be classified as a bleeding event.

Type 5: Fatal bleeding

Fatal bleeding is bleeding that directly causes death with no other explainable cause.

BARC fatal bleeding is categorized as either definite or probable as follows:

Type 5a:

Probably fatal bleeding: bleeding that is clinically suspicious as the cause of death, but the bleeding is not directly observed and there is no autopsy or confirmatory imaging

Type 5b:

Definite fatal bleeding: bleeding that is directly observed (by either clinical specimen [blood, emesis, stool, etc.] or imaging) or confirmed on autopsy

The site of fatal bleeding is specified as intracranial, gastrointestinal, retroperitoneal, pulmonary, pericardial, genitourinary, or other.

Bleeding Academic Research Consortium (BARC) indicates fatal bleeding is meant to capture deaths that are directly due to bleeding with no other cause. The time interval from the bleeding event to the death should be considered with respect to likely causality, but there is no specific time limit proposed. Bleeding that is contributory but not directly causal to death is not classified as fatal bleeding but may be categorized as other forms of bleeding. Bleeding that leads to cessation of antithrombotic or other therapies may be contributory but again would not be classified as fatal bleeding.

Confirmation of the stroke/TIA diagnosis

In the event of a stroke/TIA the diagnosis must be confirmed by the following:

- Neurologist or neurosurgical specialist
- Neuroimaging (CT, or MR,and CTA (computed tomography angiography) or MRA (magnetic resonance angiography)

Requirements post confirmed stroke/TIA

In order to determine device placement, LAA flow parameters, and clinical outcomes of the stroke/TIA, the following must be completed:

- TOE
- Modified Rankin Scale assessment 90 days following confirmed stroke/TIA

Thrombus

If a thrombus is detected post- procedure, complete an Adverse Event eCRF. The following data will be collected on the CRFs:

- Medication regime at time thrombus
- Medication(s) used to treat thrombus
- Imaging performed to monitor thrombus and frequency of monitoring
- Timeframe for resolution

8.1.1 Serious adverse device effect

A serious adverse device effect is a serious adverse event related to the device or procedure which resulted in any of the consequences characteristic of a serious adverse event, or that might have led to any of these consequences if suitable action had not been taken or intervention had not been made or if circumstances had been less opportune.

8.1.2 Assessing, recording and reporting serious adverse events, serious adverse device effects, and device deficiencies/complaints

Safety surveillance within this study and the safety reporting performed both by the investigator and Sponsor starts as soon as the dilator/device delivery system has been introduced. The safety surveillance and the safety reporting will continue until the last visit has been performed, the subject is deceased, the subject/investigator concludes their participation into the study or the subject/investigator withdraws the subject from the study.

All serious adverse events (including deaths), serious adverse device effects, and device deficiency information (if applicable) will be collected throughout the clinical study and reported to the Sponsor. Investigators will record all serious adverse events, serious adverse device effects, and deaths on the appropriate eCRF. Device deficiencies will be reported per country reporting timeline requirements to the Sponsor outside of the database on an Event Report Form (**Appendix I**).

For any serious adverse event (including deaths) and any serious adverse device effect, the investigator shall notify the Sponsor immediately of the investigator's awareness of the event and provide the Sponsor with all necessary documentation needed. Device deficiencies will be reported to the Sponsor outside of the database on an Event Report Form (**Appendix I**), as soon as possible after first learning of the event.

Records relating to the subject's subsequent medical course must be maintained and submitted (as applicable) to the Sponsor until the event has subsided or, in case of permanent impairment, until the event stabilizes and the overall clinical outcome has been ascertained. Additional

information may be requested, when required, by the Sponsor in order to support the reporting of serious adverse events to the CEC, regulatory authorities and/or other authorities.

Serious adverse events and serious adverse device effects should be monitored by the investigator until resolved. The status of the subject's condition should be documented at each follow-up visit.

Events reportable to the Sponsor, EC, CAs and regulatory authorities (as applicable) include:

- All serious adverse device effects
- All serious adverse events (including deaths and whether or not the event is considered device or procedure related)
- Device deficiencies (if applicable) that could have led to a serious adverse device effect f either suitable action had not been taken; if intervention had not been made or if circumstances had been less fortunate

The Sponsor will ensure that all events and device deficiencies are reported to the relevant authorities per country specific regulations.

8.1.3 Subject death

All subject deaths with all necessary documentation needed are to be reported to the Sponsor immediately upon the investigator's awareness of the event. The Sponsor must report the death (classified as a serious adverse event) to the NCAs where the study has commenced no later than 2 calendar days after awareness of the event. An Adverse Event eCRF should be completed and include additional detail surrounding the death and cause of death. The principal investigator should also record observed device deficiencies and assessment, if applicable.

8.2 Device Deficiency/Complaint Handling

During the trial, the investigator will be responsible for reporting all device deficiencies per country reporting timeline requirements. A device deficiency is defined as an inadequacy of a medical device with respect to its identity, quality, durability, reliability, safety or performance and includes malfunctions, and inadequate labeling.

If the device deficiency involves a serious adverse event, the investigator shall notify the Sponsor immediately of the investigator's awareness of the event and provide the Sponsor with all necessary documentation needed.

If the device deficiency does not involve a serious adverse event, the principal investigator should notify the SJM Product Surveillance Department by emailing the information about the device deficiency to the local country office or to: complaints_amplatzer@sjm.com or calling +1 6517565400 as soon as possible after becoming aware of the device deficiency. This information will be collected outside of the database on an event report form located in **Appendix I**. The event report form should be completed by a local SJM representative or the investigator. Please contact the local SJM representative to coordinate product returns as applicable.

St. Jude Medical will manage all device deficiencies related to the identity, quality, durability, reliability, safety or performance of the marketed study device and reporting to the appropriate regulatory bodies.

9. DATA MANAGEMENT

Overall, the Sponsor will be responsible for data handling. The Sponsor and/or its affiliates will be responsible for compiling and submitting all required reports to governmental agencies.

Data will be analyzed by the Sponsor and may be transferred to the Sponsor's locations outside of Europe and/or any other worldwide regulatory authority in support of a market-approval application.

St. Jude Medical respects and protects personally identifiable information that is collected or maintained. As part of its commitment, SJM is certified to the U.S. - European Union Framework and U.S. – Swiss Safe Harbor Framework Agreements regarding human resources and subject clinical trial personal information. The privacy of each subject and confidentiality of his/her information will be preserved in reports and when publishing any data. Confidentiality of data will be observed by all parties involved at all times throughout the clinical study. All data will be secured against unauthorized access.

Electronic CRFs will be used in this study, as noted below and in the data management plan. Informed consent documents will be translated to each country's language, as applicable. If additional documentation is required for any reason (e.g. procedural notes for an adverse event), it is to be appropriately redacted/de-identified prior to being sent to SJM. Source documents will be collected and translated, as needed, for CEC meetings, reporting, etc.

The principal investigator or institution will provide direct access to source data during and after the clinical study for monitoring, audits, EC review and regulatory authority inspections.

9.1 Data Management Plan

A detailed data management plan (DMP) will be established to ensure consistency of the collected data. This document will include procedures used for data review, database cleaning, and issuing and resolving data queries. If appropriate, the DMP may be updated throughout the study duration. All revisions will be tracked and document controlled.

Electronic CRF data will be captured in a validated electronic database management Oracle Clinical system hosted by SJM. Only authorized site personnel will be permitted to enter the data through the electronic data capture (EDC) system deployed by SJM. An electronic audit trail will be used to track any subsequent changes of the entered data.

9.2 Document and Data Control

9.2.1 Document and data traceability

The investigator will ensure accuracy, completeness, legibility and timeliness of the data reported to the Sponsor on the eCRFs and in all required reports.

9.2.2 Recording data

Source documents will be created and maintained by the investigational site throughout the clinical study. Data reported on the eCRFs will be derived from, and be consistent with, these source documents, and any discrepancies will be explained in writing.

The eCRFs will be signed and dated (validated eCRF) by the authorized site personnel, as specified in the Data Management Plan.

10. MONITORING

It is the responsibility of SJM as the Sponsor of the study to ensure the study is conducted, recorded, and reported according to the approved protocol, subsequent amendment(s), applicable regulations, and guidance documents. Monitoring will be conducted according to the SJM Clinical Monitoring standard operating procedure.

Centralized monitoring will occur through routine internal data review. This monitoring is designed to identify missing and inconsistent data, data outliers, and potential protocol deviations that may be indicative of site non-compliance. On-site monitoring may occur at the discretion of the Sponsor if significant concerns arise. The investigator shall make subject and study records available to the clinical monitor for monitoring.

11. REGULATORY INSPECTIONS

The investigator and/or delegate should contact SJM immediately upon notification of a governmental agency inspection at the site. A clinical monitor or designee will assist the investigator and/or delegate in preparing for the audit.

An investigator, or any person acting on behalf of such a person with respect to the study, will permit authorized governmental agency employees, at reasonable times and in reasonable manner, to inspect and copy all records relating to the study.

An investigator will permit authorized governmental agency employees to inspect and copy records that identify subjects, upon notice that governmental agency has reason to suspect that adequate informed consent was not obtained, or that reports required to be submitted by the investigator, to the Sponsor or EC have not been submitted or are incomplete, inaccurate, false or misleading.

12. STATISTICAL METHODS

No formal hypothesis will be tested for this observational post-market study. However, point estimates and exact 95% confidence intervals will be provided for the following rates in the study:

- Procedural serious adverse events (0 - 7 days) post procedure or before hospital discharge whichever latest) will be summarized for all subjects as well as stratified by imaging modality used for implanting the device (TOE and ICE)
- Late serious adverse events through 2 years
- Occurrence of ischemic stroke , systemic embolism and cardiovascular death through 2 years
- Occurrence of bleeding (including hemorrhagic stroke) events through 2 years

Data collected on the following objectives will also be reported:

- Technical success (secondary objective)
- Procedural success (secondary objective) - This objective will be summarized for all subjects as well as stratified by imaging modality used for placement of the device (TOE and ICE)
- OAC usage through 2 years (secondary objective)

- Serious Adverse Events – this may include, but is not limited to:
    - Significant bleeding events (e.g. cardiac tamponade, central nervous system (CNS) bleeding, and any bleeding related to the device or procedure that necessitates an operation)
    - Endocarditis
    - Device embolization (summarized by those that can be snared, and those that require surgical intervention)
    - Thrombus on device
    - All strokes
    - Transient ischemic attacks
    - Systemic embolism
    - Cardiovascular death
- Closure at procedure and 1-3 months post implant
    - The echo core lab's adjudication of residual flow will be used for this analysis. The following guidelines will be used:
        - None – No color flow jet in or out of the LAA
        - Small leak: < 3 mm diameter jet by color flow Doppler
        - Medium leak: 3-5 mm diameter by color flow Doppler
        - Large leak: > 5 mm diameter jet by color flow Doppler

Other variables may be summarized as deemed appropriate. The numerator for all rates will be the number of subjects who experience the corresponding event. The denominator for all rates will be subjects who experience the corresponding event plus all other subjects who achieve 2-years of follow-up.

Version 9.0 or higher of the SAS® statistical software package or other widely accepted statistical software will be used to provide all statistical analyses. Unless otherwise stated, attempts will not be made to adjust for multiple comparisons.

13. ADVISORY COMMITTEES

13.1 Steering Committee

The Steering Committee (SC) will be composed of medical practitioners who are experts in the field of AF and LAA occlusion. The committee will serve as an advisory board during the course of the study as well as after its completion. Specific responsibilities of the members will depend upon the consulting expertise needed by the Sponsor. At any time during the course of the study, the SC may offer opinions or make formal recommendations concerning aspects of the study that impact subjects. Additionally, the SC may act as an advisory panel for questions that arise during the course of the study. It will be the responsibility of the SC to write a publication policy and along with SJM, review manuscripts and conference presentations.

13.2 Clinical Events Committee

The clinical events committee (CEC) is an independent physician group that will review and adjudicate serious adverse events that occur over the course of the study. The CEC members will not be site investigators in the study. Responsibilities and membership of the CEC will be described in a CEC charter. Board membership may include cardiologists, electrophysiologists, neurologists and neuroradiologists.

The primary responsibilities of the CEC over the course of the study are to review and refine serious adverse event definitions, and to review and adjudicate serious adverse events.

13.3 Echocardiography Core Laboratory

An independent echocardiography core laboratory (echo core lab) will analyze all TOEs, TTEs, and ICE images during the trial as required per the echo core lab protocol **(Appendix E**). The echo core lab will assess residual flow into the left atrial appendage around the device in the following manner:

- None – No color flow jet in or out of the LAA
- Small leak: < 3 mm diameter jet by color flow Doppler
- Medium leak: 3-5 mm diameter by color flow Doppler
- Large leak: > 5 mm diameter jet by color flow Doppler

14. DOCUMENT RETENTION

After the termination of the study, the principal investigator will maintain all clinical study documents on file at the site for the minimum number of years required per local laws.

15. AMENDMENTS TO THE CLINICAL PROTOCOL

Study related documents such as, the Investigator's Brochure (IB) (**Appendix D**), protocol, eCRFs (**Appendix H)** informed consent form (**Appendix G**) and other subject information, or other clinical study documents will be amended as needed throughout the clinical study, and a justification statement will be included with each amended section of a document. Proposed amendments to the protocol will be agreed upon between the Sponsor and the coordinating investigator (if applicable).
The amendments to the protocol and the subject's informed consent form will be notified to, or approved by, the EC and regulatory authorities, if required. The version number and date of amendments will be documented (**Appendix B**).

The amendment will identify the changes made, the reason for the changes and if it is mandatory or optional to implement the amendment. Any amendment affecting the subject requires that the subject be informed of the changes, and a new consent be signed and dated at the subject's next follow up.

16. OUTSOURCING OF SPONSOR DUTIES AND FUNCTIONS

The Sponsor does not anticipate transferring any duties and functions related to the clinical study, including monitoring, to an external organization (such as a contract research organization or individual contractor). If outsourcing does occur, the ultimate responsibility for the quality and integrity of the clinical study will reside with the Sponsor. All requirements applying to the Sponsor will also apply to

the external organization inasmuch as this organization assumes the clinical study related duties and functions of the Sponsor.

17. INVESTIGATION SUSPENSION OR TERMINATION

17.1 Premature Termination

The Sponsor reserves the right to stop the study at any stage, with appropriate written notice to the investigator.

Possible reasons for early termination of the study by the Sponsor, either at local, national or international level, may include, but are not limited to:

- Sponsor’s decision
- Request from regulatory authorities
- Request of EC(s)
- Concern for subject safety and welfare
- Failure to secure subject informed consent prior to any protocol activity
- Failure to report unanticipated adverse device effects within 72 hours to SJM and the EC
- Repeated non-compliance with this clinical protocol or the clinical trial agreement
- Inability to successfully implement this clinical protocol
- Violation of the Declaration of Helsinki (refer to Appendix C)
- Violation of applicable national or local laws and regulations
- Falsification of data, or any other breach of ethics or scientific principles

In such events, the study will be terminated according to applicable regulations. The investigator may also discontinue participation in the clinical study with appropriate written notice to the Sponsor. Should either of these events occur, the investigator should provide a written statement as to why the premature termination has taken place, and notify the EC and/or the CA (if applicable). Follow-up for all enrolled subjects will be per center standard of care.

A principal investigator, EC, or regulatory authority may suspend or prematurely terminate participation in a clinical study at the investigational sites for which they are responsible.

17.2 Study Conclusion

The study will be concluded when:

- All follow up visits have been completed and subjects withdrawn AND
- All sites are closed AND
- The final report generated by SJM has been provided to sites or SJM has provided formal documentation of study closure

18. PUBLICATION POLICY

The results of the clinical study are planned to be submitted for publication. If a principal investigator will be involved in publishing the data, a publication agreement will be signed between the PI and Sponsor either as a separate publication agreement or within the clinical trial agreement.

The investigator or site may not publish any information that the Sponsor believes to be confidential information. The publication of the initial results of the Amulet observational post-market study shall be subject to review and release of the Sponsor's publication committee, which shall confer with the site regarding such publication.

For more information on publication guidelines, refer to the International Committee of Medical Journal Editors (ICMJE) on www.icmje.org. This study will also be posted on ClinicalTrials.gov and results will be posted on ClinicalTrials.gov as required.

19. BIBLIOGRAPHY

1. Ostermayer SH, Reisman M, Kramer PH, Matthews RV, Gray WA, Block PC, Omran H, Bartorelli AL. Percutaneous left atria appendage transcatheter occlusion (PLAATO System) to prevent stroke in high risk subjects with non-rheumatic atrial fibrillation: results from the international multi-center feasibility trials. J Am Col Cardiol. 2005; 46(1):9-14.
2. Blackshear JL, Odell JA. Appendage obliteration to reduce stroke in cardiac surgical subjects with atrial fibrillation. Ann Thorac Surg. 1996; 61:755-9.
3. Go AS, Hylek EM, Chang Y, Phillips KA, Henault LE, Capra AM, Jensvold NG, Selby JV, Singer DE. Anticoagulation therapy for stroke prevention in atrial fibrillation: how well do randomized trials translate into clinical practice? JAMA. 2003; 290(20):2685-92.
4. Stollberger C, Schneider B, Finsterer J. Elimination of the left atrial appendage to prevent stroke or embolism? Anatomic, physiologic, and pathophysiologic considerations. Chest 2003; 124(6):2356-62.
5. Miller VT, Rothrock JF, Pearce LA, Feinberg WM, Hart RG, Anderson DC, Ischemic stroke in subjects with atrial fibrillation: effect of Aspirin according to stroke mechanism. Stroke prevention in atrial fibrillation investigators. Neurology. 1993; 43(1):32-6.
6. Alizadeh A, Maeki M, Bassiri H, Alasti M, Emkanjoo Z, Haghjoo M, Arya A, Bagherzadeh A, Faze Lifar A, Sadr-Ameli MA. Evaluation of atrial thrombus formation and atrial appendage function in subjects with pacemaker by transesophageal echocardiography. Pacing Clin Electrophysiol. 2006; 29(11):1251-4.
7. Agmon Y, Khandheria BK, Gentile F, Seward JS. Echocardiographic assessment of the left atrial appendage. J Am Coll Cardiol. 1999; 34; 1867-1877.
8. Ezekowitz MD, James KKE, Nazarian SM, Davenport J, Broderick JP, Gupta Sr, Thadani V, Meyer ML, Bridgers SL. Silent cerebral infarction in subjects with nonrheumatic atrial fibrillation. The veterans affairs stroke prevention in nonrheumatic atrial fibrillation investigators. Circulation. 1995; 92(8):2178-82.

9. Gage BF, Waterman AD, Shannon W, Boechler M, Rich MW, Radford MJ. Validation of clinical classification schemes for predicting stroke: results from the national registry of atrial fibrillation. JAMA. 2001; 285(22):2864-70.
10. Andersen LV, Vestergaard P, Deichgraeber P, Lindholt JS, Mortensen LS, Frost L. Warfarin for the prevention of systemic embolism in subjects with nonvalvular atrial fibrillation: a meta-analysis. Heart. 2008; 94(12):1607-13.
11. Hart RG, Pearce LA, Aguilar MI, Adjusted-dose warfarin versus aspirin for preventing stroke in subjects with atrial fibrillation. Ann Intern Med. 2007; 147(8):590-2.
12. Eikelboom JW, Wallentin L, Connolly SJ, Ezekowitz M, Healey JS, Oldgre J, Yang S, Alings M, Kaatz S, Hohnloser SH, Diener HC, Franzosi MG, Huber K, Reilly P, Varrone J, Yusuf S. Risk of bleeding with two doses of dabigatran compared with warfarin in older and younger patients with atrial fibrillation: an analysis of the randomized evaluation of long-term anticoagulant therapy (RE-LY) Trial. Circulation. 2011; 123(21): 2363-2372.
13. Patel MR, Mahaffey KW, Garg J, Pan G, Singer DE, Hacke W, Breithardt G, Halperin JL, Hankey GJ, Piccini JP, Becker RC, Nessel CC, Paolini JF, Berkowitz SD, Fox KAA, Califf RM, and the ROCKET AF steering committee for the ROCKET AF investigators. Rivaroxaban versus warfarin in nonvalvular atrial fibrillation. N Engl J Med 2011; 365:883-891.
14. Granger CB, Alexander JH, McMurray JJ, Lopes RD, Hylek EM, Hanna M, Al-Khalidi HR, Ansell J, Atar D, Avezum A, Bahit MC, Diaz R, Easton JD, Ezekowitz JA, Flaker G, Garcia D, Geraldes M, Gersh BJ, Golitsyn S, Goto S, Hermosillo AG, Hohnloser SH, Horowitz J, Mohan P, Jansky P, Lewis BS, Lopez-Sendon JL, Pais P, Parkhomenko A, Verheugt FW, Zhu J, Wallentin L; ARISTOTLE Committees and Investigators. Apixaban versus warfarin in patients with atrial fibrillation. N Engl J Med. 2011 Sep 15;365(11):981-92.
15. Giugliano RP, Ruff CT, Braunwald E, Murphy SA, Wiviott SD, Halperin JL, Waldo AL, Ezekowitz MD, Weitz JI, Špinar J, Ruzyllo W, Ruda M, Koretsune Y, Betcher J, Shi M, Grip LT, Patel SP, Patel I, Hanyok JJ, Mercuri M, Antman EM; ENGAGE AF-TIMI 48 Investigators. N Engl J Med. 2013 Nov 28;369(22):2093-104.
16. Holmes DR, Reddy VY, Turi ZG, Doshi SK, Sievert H, Buchbiner M, Mullin CM, Sick P; PROTECT AF Investigators. Percutaneous closure of the left atrial appendage versus warfarin therapy for prevention of stroke in patients with atrial fibrillation: A randomized non-inferiority trial. Lancet 2009; 374:534-42.
17. Reddy VY, Holmes D, Doshi SK, Neuzil P, Kar S. Safety of percutaneous left atrial appendage closure: results from the Watchman left atrial appendage system for embolic protection in patients with AF (PROTECT AF) clinical trial and the continued access registry. Circulation. 2011; 123:417-24.
18. Holmes DR, Kar S, Price MJ, Whisenant B, Sievert H, Doshi SK, Huber K, Reddy VY. Prospective randomized evaluation of the Watchman left atrial appendage closure device in patients with atrial fibrillation versus long-term warfarin therapy: The PREVAIL trial. J Am Coll Cardiol. 2014; 64:1-12.

19. Reddy VY, Doshi SK, Sievert H, Buchbinder M, Neuzil P, Huber K, Kar S, Halperin J, Whisenant B, Swarup V, Holmes D. Long term results of PROTECT AF: The mortality effects of left atrial appendage closure versus warfarin for stroke prophylaxis in AF. Heart Rhythm Society 2013 Scientific Sessions; May 9, 2013; Denver, Co.

20. Gangireddy Sr, Halperin JL, Fuster V, Reddy VY. Percutaneous left atrial appendage closure for stroke prevention in patients with atrial fibrillation; an assessment of net clinical benefit. Eur Heart J. 2012; 33:2700-8.

21. Park HW, Bethencourt A, Sievert H, Santoro G, Meier B, Walsh K, Lopez-Minquez JR, Meerkin D, Valdes M, Ormerod O, Leithauser B. Left atrial appendage closure with AMPLATZER cardiac plug in atrial fibrillation: Initial European experience. Catheter Cardiovasc Interv. 2011; 77:700-6.

22. Urena M, Rodes-Cabau J, Freixa X, Saw J, Webb JG, Freeman M, Horlick E, Osten M, Chan A, Marquis JF, Champagne J, Ibrahim R. Percutaneous left atrial appendage closure with the AMPLATZER cardiac plug device in patients with non-valvular atrial fibrillation and contraindications for anticoagulation therapy. J Am Coll Cardiol. 2013; 62:96-102.

23. Meerkin D, Butnaru A, Dratva D, Bertrand OF, Tzivoni D. Early safety of the AMPLATZER cardiac plug for left atrial appendage occlusion. Int J Cardiol. 2013; 168:3920-5.

24. Nietlspach F, Gloekler S, Krause R, Shakir S, Schmid M, Khattab AA, Wenaweser P, Windecker S, Meier B. Amplatzer left atrial appendage occlusion single center 10-year experience. Catheter Cardiovasc Interv. 2013; 82:283-9.

25. Bartus K, Han FT, Bednarek J, Myc J, Kapelak B, Sadowski J, Lelakowski J, Bartus S, Yakubov SJ, Lee RJ. Percutaneous left aptrial appendage suture ligation using the LARIAT device in patients with atrial fibrillation: initial clinical experience. J Am Coll Cardiol. 2013; 62:108-18.

26. Camm AJ, Lip GY, De Caterina R, Savelieve I, Atar D, Hohnloser SH, Hindricks G, Kirchof P: ESC Committee for Practice Guidelines (CPG). The 2012 focused update of the ESC guidelines for the management of atrial fibrillation. Developed with the special contribution of the European Heart Rhythm Association. Eur Heart J. 2012; 33-2719-47.

27. Saw J, Lempereur M. Percutaneous left atrial appendage closure procedural techniques and outcomes. JACC: Cardiovascular Interventions Vol. 7 No 11 2014.

28. Park JW, Bethencourt A, Sievert H, Santoro G, Meier B, Walsh K, Lopez-Minquez JR, Meerkin D, Valdés M, Ormerod O, Leithäuser B. Left atrial appendage closure with AMPLATZER cardiac plug in atrial fibrillation: Initial European experience. Catheterization and Cardiovascular Interventions, 2011; 77: 700–706.

29. Walsh K, Sievert H, Lickfett L, Omran H, Hennen B, Schraeder R, Garcia E, Park JW, Schillinger W, Lopez-Minguez JR, Valdes M, Ormerod O, Neuzil P, Hildick-Smith D, Lezaun R: Left atrial appendage closure with the AMPLATZER cardiac plug: Results of the European post-market observational study. Presented at EURO PCR May 2012.

30. Sievert H, Park JW, Schillinger W, Lickfett L, Lopez-Minquez JR, Omran H, Walsh K, Ormerod O, Neuzil P, Schraeder R, Hildick-Smith, D: Long-term follow-up from a left atrial appendage

occlusion European multicentre post market observational study. Presented at EURO PCR May 2014.

31. Lam SC, Bertog S, Gafoor S, Vaskelyte L, Boehm P, Ho RW, Franke J, Hofmann I, Sievert H. Left atrial appendage closure using the amulet device: An initial experience with the second generation Amplatzer cardiac plug. Catheter Cardiovasc Interv. 2014.

32. Freixa X, Abualsaud A, Chan J, Nosair M, Tzikas A, Garceau P, Basmadjian A, Ibrahim R. Left atrial appendage occlusion: initial experience with the AMPLATZER™ Amulet™. Int J Cardiol. 2014 Jul 1;174(3):492-6.

33. Tzikas A, Shakir S, Gafoor S, Omran H, Berti S, Santoro G, Kefer J, Landmesser U, Nielsen-Kudsk JE, Cruz-Gonzalez I, Sievert H, Tichelbäcker T, Kanagaratnam P, Nietlispach F, Aminian A, Kasch F, Freixa X, Danna P, Rezzagh M, Vermeersch P, Stock F, Stolcova M, Costa M, Ibrahim R, Schillinger W, Meier B, Park JW. Left atrial appendage occlusion for stroke prevention in atrial fibrillation: multicentre experience with the AMPLATZER Cardiac Plug. EuroIntervention. 2015 Jan 22;10(10).

34. Tzikas A, Holmes DR, Gafoor S, Ruiz CE, Blomström-Lundqvist C, Diener HC, Cappato R, Kar S, Lee RJ, Byrne R, Ibrahim R, Lakireddy D, Soliman O, Näbauer M, Schneider S, Brachman J, Saver JL, Tiemann K, Sievert H, Camm J, Lewalter T. Percutaneous left atrial appendage occlusion: the Munich consensus document on definitions, endpoints and data collection requirements for clinical studies. Draft LAAO Consensus Document. To be submitted to EuroHeart Journal. March 2015.

35. Hicks KA, Hung HM, Mahaffey KW, Mehran R, Nissen SE, Stockbridge NL, Targum SL, Temple R on behalf of the Standardized Data Collection for Cardiovascular Trials Initiative (2014). Standardized Definitions for Cardiovascular and Stroke Endpoint Events in Clinical Trials. Draft Definitions for CDISC. August 24, 2014.

36. Mehran R, Rao SV, Bhatt DL. Standardized bleeding definitions for cardiovascular clinical trials: A consensus report from the Bleeding Academic Research Consortium. Circulation 2011; 123(23):2736–47.

Appendix A: Abbreviations

Abbreviation	Term
ACP	Amplatzer Cardiac Plug
AE	Adverse Event
AF	Atrial Fibrillation
AP	Asia-Pacific
ANZ	Australia-New Zealand
CA	Competent Authority
CE	Conformité Européenne
CEC	Clinical Events Committee
CP	Clinical Protocol
CRF	Case Report Form
CNS	Central Nervous System
CT	Computed Tomography
DMP	Data Management Plan
EC	Ethics Committee
ECG	Electrocardiogram
eCRF	Electronic Case Report Form
EDC	Electronic Data Capture
EMEA	Europe, Middle East, Africa
EP	Electrophysiologist
IB	Investigator's Brochure
IC	Interventional Cardiologist
ICMJE	International Committee of Medical Journal Editors
IFU	Instructions For Use
INR	International Normalized Ratio
ISO	International Organization for Standardization
LAA	Left Atrial Appendage
LAAO	Left Atrial Appendage Occlusion
MRI	Magnetic Resonance Imaging
mRS	Modified Rankin Scale
NCA	National Competent Authority
NVAF	Non-Valvular Atrial Fibrillation
OAC	Oral Anticoagulant
PI	Principal Investigator
SADE	Serious Adverse Device Effect
SAE	Serious Adverse Event
SC	Steering Committee St.
SJM	Jude Medical, Inc.
TIA	Transient Ischemic Attack
TOE	Transoesophageal Echocardiography
WMA	World Medical Association

Appendix B: Protocol Revision History

Revision History				
Amendment Number	Version	Date	Rationale	Details
Initial Release - N/A	A	See Windchill (internal document control system)	Initial Document Release	Initial Release

Appendix C: Declaration of Helsinki

The 2013 version of the Declaration of Helsinki is available at: http://www.wma.net/en/20activities/10ethics/10helsinki/. Please check the website during the course of the study for updated revisions.

Appendix D: Investigator's Brochure (IB)

The IB for the Amulet device provides safety and performance data from pre-clinical and clinical studies. The IB will be updated throughout the course of the clinical study as significant new information becomes available (e.g. a significant change in risk, etc.).

The principal investigators, coordinating investigators, national investigators, and medical advisors will acknowledge the receipt of the IB and all subsequent amendments, and will keep all information confidential.

The Investigator's Brochure will be kept under separate cover and is available upon request.

Appendix E: Echocardiography Core Laboratory Protocols

<u>Transoesophageal Echocardiagraphy (TEE) Protocol</u>

ALL views to be provided in the following sequence:

I. TEE Scanning Protocol

The following TEE protocol is recommended to ensure uniformity across clinical study sites. TEE studies are performed at baseline, during the procedure, at the 1-3 month and applicable follow-up/interim visits. The TEE study should image the left atrium (LA) and left atrial appendage (LAA) in multiple planes (i.e., 0°- ~110°). The purpose of the TEE is to obtain information regarding: a) LA/LAA thrombus; b) LAA ostium measurements; c) length distal to the LAA ostium; d) flow into the LAA and e) to assess for procedural complications, such as device migration, pericardial effusion, etc.

II. Adjustment of the Ultrasound Instrument:

a. Adjust transmission power (gain) to minimize/eliminate “blooming” of specular reflectors.
b. Adjust the time gain compensation in such way that the image exhibits uniform brightness.
c. Adjust the color Doppler gain below the point where random color noise appears.
d. Set pulse repetition frequency with Nyquist velocity for CFD at ~35-45 cm/sec.
e. Use a velocity variance map with a “medium” color filter; set color tissue priority at a level that avoids overlay of color signals onto tissue structures.
f. Perform TEE at 5-7.5 MHz, with all other settings as mentioned above.
g. Perform both longitudinal and transverse plane sweeps to ensure full coverage of LA/LAA.
h. Record 3-5 consecutive beats for all cine-loop images; record at least 3-5 consecutive beats for all Doppler velocity tracings – record these as still frames (provide 2-3 still frames) at 100mm/s sweep speed.
i. Ensure that the ECG is recorded for all cine-loops and still-frame images.

III. TEE Imaging Study to be Obtained in the Following Views:

a. The 5-chamber view (Omniplane 0°) with the aortic valve in the center of the screen and the LA in the right upper corner.
   - Standard 2D imaging.
   - Zoom mode of LA and LAA; place color flow Doppler (CFD) around device to document communication between LA and LAA (post procedure only).
   - Zoom mode of LA and LAA; align Pulse Wave (PW) Doppler sample volume to record LAA-LA blood flow. The PW sample should be placed 0.5 to 1 cm from the ostium into the LAA.

- 2D imaging of LV at mid-papillary muscle level (short axis), from gastric view.

b. The high LA/LAA view (Omniplane ~60°)

Optional views (these may be difficult to obtain in some subjects):

- Zoom mode of LA and LAA; place CFD around device to document communication between LA and LAA (post procedure only).
- LA/LAA views; align PW Doppler sample volume to record LAA-LA blood flow. The PW sample should be placed 0.5 to 1 cm from the ostium into the LAA.

c. The longitudinal imaging plane (Omniplane ~80-110°) with LA/LAA in field of view.

- Zoom mode of LA and LAA; place CFD around device to document communication between LA and LAA (post procedure only).
- Zoom image of LA-LAA; align PW Doppler sample volume to record LAA blood flow. The PW sample should be placed 0.5 to 1 cm from the ostium into the LAA.

IV. Technical Notes for Completion of Echocardiographic Studies

a. Study subjects are to be identified only by study site and study subject number. This information will be entered in the ultrasound system patient data section in adherence to applicable privacy laws. <u>No subject names should be displayed in the submitted studies.</u>

b. Two-dimensional imaging to be performed in harmonic mode; the ECG tracing should be displayed on all images (still frames and cine-loops), clearly showing P wave/QRS morphology during the echocardiographic study. <u>Avoid recording extra systolic (PVC) beats</u>.

c. For all spectral Doppler velocities, please use a display speed of 100 mm/sec, and optimize the velocity profile by moving the baseline up or down and showing the maximal velocity obtained at the top of the scale.

d. Clinical sites should retain a digital copy of the echocardiographic study in DICOM and the study should be analyzed locally. The Echocardiography Core Laboratory WILL NOT return submitted studies. An echocardiographic analysis WILL NOT be provided by the Echocardiography Core Laboratory to the clinical sites. The Echocardiography Core Laboratory Report will only be used for the purposes of the Amulet study and NOT for diagnosis and/or clinical management of subjects enrolled in the Amulet study.

V. Variables that can be Recorded by the Study Sites for optimum imaging

a. Thrombus in the LA and/or LAA

b. LAA ostium measurements

c. Length distal to the LAA ostium

d. Spontaneous Echo Contrast (SEC) in the LA

e. Thrombus in the RA and/or RAA

f. Spontaneous Echo Contrast (SEC) in the RA
g. LAA-LA communication/flow (see E below)
h. Left Ventricular Ejection Fraction (LVEF)
i. Presence, location, and size of any aortic plaques
j. Mitral or aortic valve stenosis or regurgitation
k. In addition the following data points may also be documented: presence of
    1. Atrial septal defect
    2. Patent foramen ovale
    3. Prosthetic valve
    4. Intracardiac thrombus
    5. Atrial septal device

VI. Measurements to be analyzed by the Echocardiography Core Lab

a. Thrombus in the LA and/or LAA
b. LAA ostium measurements
c. Length distal to the LAA ostium
d. Thrombus in the RA and/or RAA
e. Spontaneous Echo Contrast (SEC) in the LA
f. Presence, location and size of any aortic plaque
g. LAA-LA communication/flow (see VII below)

VII. Assessment of Flow in Left Atrial Appendage to be analyzed by the Echocardiography Core Lab

a. **None:** No color flow jet in or out of the LAA
b. **Small leak:** < 3 mm diameter jet by color flow Doppler or multiple leaks that are cumulatively < 3 mm diameter jet by color flow Doppler
c. **Medium leak:** 3-5 mm diameter by color flow Doppler or multiple leaks that are cumulatively 3-5 mm diameter jet by color flow Doppler
d. **Large leak:** > 5 mm diameter jet by color flow Doppler or multiple leaks that are cumulatively > 5 mm diameter jet by color flow Doppler

Transthoracic Echocardiogram (TTE) Protocol

With the subject in the left lateral position, standard transthoracic imaging should be obtained in the parasternal long-axis, parasternal short-axis, apical 4-chamber, apical 2-chamber, and apical 3-chamber views. For the evaluation of left ventricular systolic function a sweep of the entire chamber from base to apex should be imaged from the parasternal short axis view. Evaluation of all 4 valves should include color flow doppler, and continuous and pulse wave doppler interrogation. With the subject lying supine, the atrial septum should be evaluated in the subcostal long axis view by both 2D imaging alone and with color flow doppler.

Any post procedure complication such as pericardial effusion must be documented.

Appendix F: List of Clinical Investigation Sites and Ethics Committees

A list of clinical investigative sites and ECs will be kept under separate cover and is available upon request.

Appendix G: Sample Informed Consent

INFORMED CONSENT
AMPLATZER™ Amulet™ Observational Post-Market Study

STUDY TITLE AND NUMBER: AMPLATZER Amulet Observational Post-Market Study

SPONSOR: St. Jude Medical, Inc. (SJM)

PRINCIPAL INVESTIGATOR: ____

SITE NAME: ____

Participant declaration of consent for participation in the Amulet Observational Post-Market Study.

Name of participant:___________________________Date:_______________________________

Introduction

This form explains why this research study is being done and what your role will be if you decide to participate in this research study. This form also talks about the possible risks that may happen if you take part in this research study. The study is sponsored by SJM. This company manufactures medical devices to treat various medical conditions.

Please read this form, and ask your study doctor any questions you may have about the study so your questions are answered before you decide if you want to take part in the study. Please take your time and talk about this information with your family, friends, or family doctor.

This consent form may contain some words that you do not understand. It is important that you understand what is in this form. It will explain the different activities you will be asked to do or participate in if you take part in the study and what the risks might be; whether or not you do take part is entirely your choice. Please ask the study doctor or the study staff to explain any words or information that you do not understand.

If you decide you want to take part in the study, you will be asked to sign the consent section before any study-related activities are performed. By signing this form you are telling us that you:

- Understand what you have read
- Consent to take part in the study
- Consent to have the tests and treatments that are described
- Consent to the use of your personal and health information as described.

Taking part in this study is entirely voluntary. If you don't wish to take part, you don't have to. You will receive the best possible care whether or not you take part in the study. Refusing participation will not involve any penalty or loss of benefit. If you decide to take part in this study, you must sign your name at the end of this form. No study activity can be performed until you sign this form.

What is the purpose of this study?
The purpose of the Amulet observational post-market study is to compile real world performance of the commercially available Amulet device in people 18 years or older with paroxysmal, persistent or permanent nonvalvular atrial fibrillation. Data collected for the study will be used to assess closure of the left atrial appendage and complications after the procedure, and the percentages of stroke and cardiovascular death. The purpose of the study is also to evaluate the success rate of the Amulet device implant, and the use of oral anticoagulants and antiplatelet medications participants take.

Why am I being invited to take part in this study?
You are being asked to take part in this study because your doctor has determined you have paroxysmal, persistent or permanent nonvalvular atrial fibrillation. Normally, electrical signals from the upper chambers of the heart (atria) travel to the lower chambers of the heart (ventricles) and cause them to beat in a regular way. During atrial fibrillation, the electrical signals in your heart are abnormal and cause the upper chambers of the heart to beat too fast and irregularly.

This irregular beating of the heart leads to a slowing of the blood flow in the upper chambers of the heart. In the left upper chamber there is a small pouch called the left atrial appendage (LAA). Slowing of blood, especially in the LAA, may cause blood clots to form. Blood clots can move from the LAA and travel to the brain, causing a stroke or transient ischemic attack (TIA), also called a ministroke. These blood clots can also travel to other parts of the body and block blood vessels.

The commercially available Amulet device could be beneficial to you because it may decrease the chance of a new blood clot forming and moving from your heart's left atrial appendage to other parts of your body. **Figure 1** shows the Amulet device in its final position in the LAA.

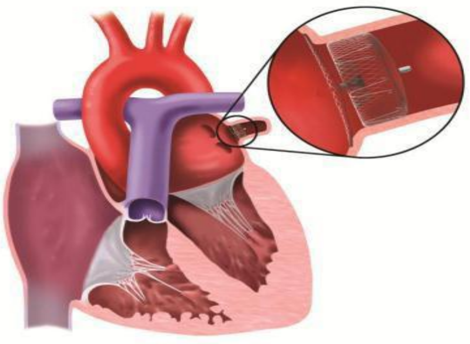

Figure 1. Placement of the Amulet device in the left atrial appendage

Your doctor has given you this form to tell you about the Amulet observational post-market study and to ask for your consent to participate. Once you understand the study and agree to take part, your signature on the last page of this form documents your willingness to participate.

<u>What is the device being tested?</u>

The Amulet device is a self-expanding device which is made from a nitinol (nickel-titanium alloy) mesh and has a lobe and a disc connected by a central waist. The lobe has small wires that help keep the device in place. The device has screw attachments at each end so it can be delivered to the heart. The device has special metal bands at each end that allow it to be seen on fluoroscopy, which is a kind of X-ray. The Amulet device has regulatory approval and is commercially available in all countries where this study is taking place.

A. Screw attachments
B. Waist of device
C. Lobe
D. Stabilizing wires
E. Disc

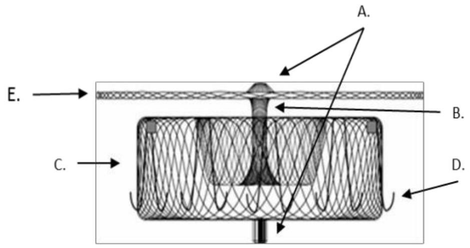

Figure 2. The Amulet device and its key components

<u>What will be requested from you if you take part in this research study?</u>

In order to evaluate the Amulet device, no testing outside of what is routine care for someone with atrial fibrillation will be done. Any tests done for the study are part of your normal care and would be done even if you were not in the study. There are no experimental procedures in this study. Your doctor will report serious adverse events to the Sponsor and the appropriate regulatory authorities. **Table 1** describes study specific activities and procedures.

Table 1. Study-Specific Activities/Procedures

Visit / Study Activity	Enrollment & Baseline	Procedure	Discharge	1-3 Months	6M Phone Visit (+/- 2 weeks)	12M (+/- 6 weeks)	24M Phone Visit (+/- 12 weeks)	Interim Visit
Informed Consent Process	X							
- History and Physical<br>- Cardiovascular and medical exam<br>- CHA_2DS_2-VASc score<br>- HAS-BLED score	X							
12-lead ECG	X							
Pregnancy Test[1]	X							
Medication Assessment	X		X	X	X	X	X	X
Modified Rankin Scale[2]	X							X[4]
Angiography		X						
TTE			X					
TOE	X[, 5]	X[3, 5]		X				X[4]
ICE		X[6]						
Adverse Event Assessment		X	X	X	X	X	X	X

[1]*Pregnancy test for women of childbearing potential*
[2]*Perform additional Modified Rank Scale assessment 90 days after a confirmed stroke*
[3]*CT can be used as additional information and performed complimentary to the TOE, per institutional standard of care*
[4]*Complete if stroke or TIA is confirmed*
[5]*The Baseline TOE is optional if a TOE is performed on the day of procedure to rule out the presence of intracardiac thrombus (including left atrial appendage thrombus) and to assess the size and shape of the LAA*
[6]*ICE may be performed for placement of the device in a sub-set of sites pre-approved by SJM*

Screening/Baseline Evaluation

This research study includes a screening/baseline evaluation to determine if you are a candidate for the study. Your doctor or other study personnel will ask you medically related questions.

You will have the following tests done after you agree to be part of this study and sign this document. These tests are common tests for someone with atrial fibrillation, and might be performed even if you do not join this study:

- Informed consent process
- History and physical
- Cardiovascular and medical history
- CHA_2DS_2-VASc – a set of questions used to assess the level of risk for stroke
- HAS-BLED scale – a set of questions used to assess the level of risk for bleeding events

- 12-lead electrocardiogram (ECG) - used to measure the electrical activity of the heart by placing electrodes on the chest, arms, and legs
- Transoesophageal echocardiography (TOE) – used to show how well your heart chambers and valves are working and to detect possible blood clots in the heart, and in the sac around your heart
  - Note the Baseline TOE is optional if a TOE is performed the day of procedure
- Modified Rankin Scale – a commonly used scale for measuring the degree of disability or dependence in the daily activities of people who have suffered a stroke or other causes of neurological disability
- Pregnancy test (if you are female of child-bearing age)
- Medication assessment – you will be asked to confirm which antiplatelet and blood-thinning medications you are taking

Once all study required tests have been completed, your doctor will decide if you qualify to take part in the Amulet observational post-market study. If you do not qualify for the study, your participation will end and you will be withdrawn from the study.

Procedure
If you meet all of the inclusion and none of the exclusion criteria, you will be scheduled for the Amulet device implant procedure which takes about 1-2 hours. If you are taking antiplatelet or blood-thinning medications, your doctor will tell when you should stop them before your implant procedure.

A trained Sponsor representative may be present at the implant to answer any questions related to the Amulet device, the procedure and the study.

Conscious sedation (medicine that will make you relaxed and sleepy yet awake) or general anesthesia (medicine that will make you sleep) will be used. Your doctor will first perform a TOE of your heart to determine the size of your LAA, and to ensure there are no blood clots in your heart. Transoesophageal echocardiography (TOE) is a test that uses sound waves to create high-quality moving pictures of the heart and its blood vessels. Your doctor will not continue with the procedure if the TOE shows your LAA is not suitable for the implant or if there is a thrombus (blood clot) in your heart. Your doctor may decide you do not need the Amulet device or that another type of surgery or commercially available device would be better for you. If you meet any of the exclusion criteria during the procedure, you will be withdrawn from the study.

If your doctor determines you meet all of the inclusion criteria and none of the exclusion criteria, a small incision will be made in your groin area to insert a delivery catheter through a vein in your leg. A catheter is a sterile, flexible, hollow tube that is put into a blood vessel to let fluids in or out of your body, or to deliver medical devices to your heart. The doctor will make a small puncture through your septum (the muscular tissue that separates the two sides of the upper heart), in order to gain entry into the upper left heart chamber to place the Amulet device.

If pre-approved by the Sponsor, your doctor may use an intracardiac echocardiography (ICE) procedure to help place the device in your heart. Intracardiac echocardiography (ICE) is an imaging technique that allows physicians to get a clear picture of the inner workings of the heart. A small ultrasound probe is placed into a vein in the groin, then threaded into the area of the heart being worked on. ICE may be used as a diagnostic tool, or for guiding the physician during minimally invasive procedures inside the

heart. The ICE procedure is completely painless and involves only the use of a local anesthetic at the site of insertion.

You are considered enrolled in the study when the device delivery system is introduced into your body. An appropriately sized Amulet device will be passed through the catheter and placed in your LAA. To ensure the Amulet device is placed correctly in your LAA, your doctor will use routine measurements such as angiography and TOE. Angiography is a medical imaging technique used to visualize the inside of blood vessels and organs of the body, with special interest in the arteries, veins, and the heart chambers. This is done by injecting a radio-opaque contrast agent into the blood vessel and imaging using X-ray based techniques such as fluoroscopy. After your doctor is satisfied with the position of the device, the catheter is removed. The Amulet device will remain permanently in your heart. Over time, the tissue in your body will cover and grow around the device to further hold it in place. After the procedure, you may have some minor pain in the groin area where the catheter was inserted.

If you meet all of the inclusion and none of the exclusion criteria, and the delivery system was introduced into your body but you did not receive the device (attempt to treat), your participation will end and you will be withdrawn from the study.

Pre-Discharge Visit
You may be kept overnight for observation after the procedure. A pre-discharge transthoracic echocardiography (TTE) will be done to assess for any post procedure complications and to verify the position of the device in your heart. A TTE is another type of echocardiogram that uses high-pitched sound waves sent through a transducer (a hand-held plastic instrument which looks like a microphone) that is placed on different areas of your chest. The transducer picks up echoes of the sound waves as they bounce off the different parts of your heart and turns them into moving pictures of your heart that can be seen on a video screen. Your health and medication status will be assessed. If you are discharged on antiplatelet medication, it is recommended you take aspirin for six months and Plavix for one month. If you are discharged on warfarin, INR testing should be performed periodically to ensure your INR is in the range of 2-3. Your doctor will determine when you should discontinue antiplatelet medications or warfarin. If you discontinue any of these medications before your 6 month visit, you should tell your doctor.

Follow-up Visits
It is important to keep all follow-up appointments scheduled for you. The study requires in clinic follow-up visits about 6 weeks and 12 months after your procedure. These visits take approximately half a day. During these visits you will be asked about your health and the medications you are taking. You will also undergo a TOE at your 6 week visit to make sure your Amulet device has not moved and has closed your LAA.

The study requires telephone visits about 6 months and a minimum of 2 years and potentially 3, 4 and 5 years after your procedure. These telephone visits will take about 30 minutes. Your doctor or nurse will call you and ask about your health and the medications you are taking. If you had a stroke, you should tell your study doctor and return to the clinic about 90 days after the event.

You will be notified of your discontinuation date, which could occur a minimum of 2 or a maximum of 5 years after your procedure date depending on the duration of the study.

Interim/Unscheduled Visits
If you are seen by your doctor in addition to the recommended visits listed in **Table 1**, you will be asked about your health and the medications you are taking.
If you had a stroke or TIA, a Modified Rankin Scale assessment will be performed about 90 days after the event to assess the degree of your disability and dependence in daily activities. You are required to have a TOE if you have a stroke or TIA. You may be seen by a neurologist and additional testing may be performed. The results from the TOE will be used to make sure the Amulet device is still in the correct location and that your LAA is closed.

How long will the study last and how many will take part?
If you agree to take part in this research study, your involvement will last a minimum of 2 years and will not exceed 5 years. About 1000 people will take part in this research study at about 75 sites worldwide.

What are the possible risks and discomforts?
There are risks, discomforts, and inconveniences associated with any study to you (or to an embryo, unborn child or nursing infant if you become pregnant). These deserve careful thought. The risks associated with participation in this study include risks with the study design, complications during and after the procedure, and risks with the collection of your data. The risks are listed below but will vary from person to person. There may be other risks that are unknown at this time. You should talk with your study doctor if you have any questions.

Risks with Study Design
The Sponsor will select investigators qualified by training and experience to participate in this study. Participating sites will be selected based upon qualifications of the primary investigator. During the study, sites may be subject to quality assurance audits by the Sponsor (or designee), as well as monitoring visits to assess data integrity and study compliance. Additionally, the study will have an appointed independent Clinical Events Committee (CEC) who will review and adjudicate all reported adverse events, as well as have general study oversight of subject safety and data integrity.

Risks with Baseline and Follow-Up Testing
The potential risks associated with review of your medical record include but are not limited to:

- Who will have access to the data
- Specifying how and at what point in the research personal information will be separated from other data
- Whether the data will be retained at the conclusion of the study and for how long

These risks will be mitigated by ensuring:

- There is an adequate plan to protect identifiers from improper use and disclosure
- There is an adequate plan to destroy identifiers at the earliest opportunity
- Protected health information (PHI) will not be re-used or disclosed for another purpose

You may experience some anxiety when asked abut your health, medications, and neurological status. You may be inconvenienced by traveling to the study site for your study visits, or for having to miss work to take part in the study.

Risks and Complications with the Implant Procedure

Your doctor will explain the risks and discomforts of the implant procedure and the risks of the commercially available Amulet device.

There may be other risks or discomforts to you (or to an embryo, unborn child or nursing infant if you become pregnant) that are not known at this time.

New findings
Your doctor will discuss with you important new information that is learned during the course of this study that may affect your condition or willingness to continue to take part in this research study. You will also be informed of the reasons for changes to the clinical protocol. If you decide to withdraw or if your doctor decides it is in your best interest to withdraw due to the new findings, your doctor will explain the reasons and arrange the necessary steps for your care to continue outside of the study.

What are the risks for women of childbearing age?
To confirm to the extent medically possible that you are not pregnant, you agree to have a pregnancy test done before beginning this research study.

If you are a woman who is able to become pregnant, it is expected that you will use an effective method of birth control to prevent exposing a fetus to a potentially dangerous agent with unknown risk. If you are pregnant or currently breast feeding, you may not participate in this study. You understand that if you are pregnant, if you become pregnant, or if you are breast-feeding during this study, you or your child may be exposed to an unknown risk. Subjects who become pregnant while taking part in the study should tell their study doctor right away.

Residual Risks
The overall events from a risk perspective are similar with respect to minimally invasive implantable therapy. Complication rates are within expected ranges and the risk/benefit profile is similar to alternative therapies.

What are the possible benefits to you or others?
If you decide to take part in this study and have the Amulet device implanted, you may not need to be on long-term warfarin oral anticoagulation therapy. You may also benefit from a decrease in the risk of getting a blood clot due to atrial fibrillation, but there is no guarantee this will happen.

The information gathered in this study may benefit others by adding to the understanding of treatment options for patients with nonvavular atrial fibrillation.

If you do not want to take part in this research study, what other options are available to you?
If you choose not to participate in this study at this time, your doctor will discuss other options available to you. Treatment options may include the use of commercially available devices including the Amulet device outside of the study, surgery and/or drugs to prevent the occurrence of blood clots, which can cause stroke, or not having any treatment at all. You may also choose not to participate.

If you choose to take part in this study, what are the costs?
Your medical insurance will be billed for all standard costs for the Amulet device, the procedure and the follow-up visits, which are standard of care.

Will you receive payment for taking part in this research study?
No payment will be made to you for taking part in this research study.

What if the device needs to be removed?

In the event your Amulet device or any part has to be removed, it may be returned to SJM for analysis. Should you withdraw from this study and choose to have your Amulet device or any part of it removed, the cost will be your responsibility.

In the event of your death, your implanted Amulet device may be removed and returned to SJM for analysis. The study doctor will get your family's approval prior to removing the device.

Insurance
To compensate for financial loss due to study-related damage to health, the manufacturer has concluded legally prescribed clinical trial insurance coverage for all subjects. During the period of the clinical trial other medical treatment should only take place in consultation with the investigator, with the exception of emergency situations. Upon suspicion of study-related damage to health, you must immediately notify the treating investigator so that he can inform the insurance office on your behalf. You can expect to receive a copy of the report. The insurance is concluded with Allianz Global Corporate & Specialty.

What if you are injured because of this study?
If you suffer any injuries, illnesses, or complications as a direct result of participation in this study, conducted in accordance with the study protocol, you should contact your study doctor as soon as possible and you will be assisted with arranging appropriate medical treatment. For those expenses that are not covered by your insurance, SJM will pay the costs of reasonable and necessary treatment of injuries, undesirable side effects, or adverse reactions caused by study procedures that are not standard of care for you, or caused by a defect or malfunction of the study device and/or problems with the study procedure during the study. The reimbursement may be provided directly to you, or to <<*insert site name*>> for cost incurred in treating the study-related injury, illness or complication. No other arrangement has been made for financial payments or other forms of compensation (such as lost wages, lost time or discomfort) with respect to such injuries. If emergency medical treatment is needed, your study doctor will provide medical care to the extent permitted under the applicable national, regional, or local law. You do not waive any legal rights by signing this consent form.

During the study, if you experience any injuries, illnesses, or complications from taking part in this research study, please contact Dr. ______________at ___________.

What are your rights if you decide to take part or stop participation in this research study?
Your signature on this consent form means that you have received information about this research study and that you agree to be a part of the research study.

You may stop taking part in the research study at any time without penalty or loss of benefits to which you are otherwise entitled. If you wish to stop taking part in this research study for any reason, you should contact Dr. _____________at___________.

If you withdraw your consent during the study, the study doctor and relevant study staff will not collect additional personal information from you, although personal information already collected will be retained to ensure that the results of the research study project can be measured properly and to comply with the law. You should be aware that data collected by the Sponsor up to the time you withdraw will be part of the study results.

Your study doctor or designee will discuss with you what follow-up is required if you decide to withdraw, or are withdrawn from the research study before the study is finished. Your doctor or the

Sponsor of the study (SJM) may also stop your participation in the research study at any time, without your consent, for any reason. The Sponsor can suspend or prematurely terminate the study for safety, business purposes, or other reasons.

How will your information be kept confidential?

If you decide to take part in this study, your medical records and personal information will be kept confidential to the extent allowed by national, regional and local law. However, information from the study may be exported to countries where different data protection laws apply. The data protection laws in other countries may be less strict than those of your country.

If you decide to participate in the research study, the study Sponsor and others who work with the study, such as the study staff and Ethics Committee (EC) will see health information about you. The EC is a group of people who perform independent review of research studies as required by laws governing this type of research study. Other regulatory agencies, the EC and the Sponsor's representatives may inspect and copy your medical records.

The information collected about you may be used in several ways. Information about you and your health that might identify you may be given to others to carry out the research study. Your study doctor may use some of the information in making decisions about your care.

The Sponsor may use the information in any of the following ways:

- To analyze and make conclusions about the results of the study
- In documents sent to the government agencies throughout the world
- For reporting undesirable events to government health agencies
- To provide overall study results to other study doctors, including in publications
- To conduct new medical research studies, to reanalyze the study results in the future or to combine your information with information from other studies
- To develop new medical products and procedures, and other product-development related activities

Your name will not be provided for publications in medical journals. While using the information in these ways, the Sponsor may give study data to its affiliated companies in the U.S. or other countries. The Sponsor may also share the information with its business partners or companies it hires to provide study-related services. Information received during the research study will not be used for any mailing lists or sold to anyone for marketing purposes.
The study will not collect the initials of your name. The study will only collect the month and year of your birth. Your permission for the use, retention, and sharing of your identifiable health information will continue indefinitely.

A description of this clinical study will be available on http://www.ClinicalTrials.gov, as required by U.S. Law. This Web site will not include information that can identify you. At most, the Web site will include a summary of the results. You can search the Web site at any time.

Information about the clinical investigation will be available on another public database, if not available on http://www.ClinicalTrials.gov or if it is required by local regulations.

Who can you contact for study information?

If you have any questions about the study or taking part in this research study, or if you have suffered a study-related injury, please contact Dr. ______________at ___________.

In addition, if you have any concerns, complaints or questions about your rights as a research study participant or an injury that you believe is a study-related, please contact the EC:

Name of person at EC:
Title of person at EC:
EC phone number:
EC email, if known:

Consent and authorization for participation in this research study

Taking part in this research study is entirely voluntary. You are making a decision on whether or not to take part in the research study. Your signature indicates that you have read the information in this form and have decided to take part in the research study. You will be given a signed copy of this form to keep.

√ I have read and understood the information of this Amulet obervational post-market study and the research staff has answered all of my questions regarding the study.

√ I have had enough time to think about my participation in this Amulet obervational post-market study and I am aware that participation in this study is voluntary.

√ I realize that I may decide to refuse participation or stop participation at any time without affecting the quality of my heath care or the relationship with my physician.

√ I am free to withdraw at any time, without giving any reason and without my medical care or legal rights being affected. There will not be any penalty if I decide to withdraw from the study.

√ I understand and agree that personal information about me will be collected from my medical records and processed by SJM or any other entity involved in the study. My medical information, information collected during the study and study results may be looked at, used, and disclosed by responsible individuals from SJM, a U.S. company, and its affiliates (located in the U.S. and other countries), and other people who work for SJM to provide services related to the device and this study, regulatory authorities, the Ethics Committee and, when it is relevant to my taking part in this research. I give permission for these individuals to have access to my medical information, information collected during the study, and study results.

√ I authorize and instruct my physician(s) and the institution to release personal information about me necessary to conduct this Amulet observational post-market study.

√ I understand I am free to access my personal information and to have errors corrected.

√ My data gathered in this study may be stored (after anonymized) in a database and may be used and disclosed by SJM.

√ Data collected from the time I sign this document until the time I withdraw from the study will be used after I withdraw.

√ I will not financially benefit from this study.

√ I understand how to contact the research team.

√ My attendance at study visits is important to the study and I should follow the study doctor‘s instructions.

√ I am willing to participate in this study.

<u>Please check one option below:</u>

☐ I agree that my personal physician is informed about my participation in this study.

☐ I disagree that my personal physician is informed about my participation in this study.

Name of Participant (please print): ______________________________

Signature ______________________ Date ______________________

Name of Person Obtaining Consent (please print) ______________________________

Signature ______________________ Date ______________________

Appendix H: Case Report Forms

Case Report Forms will be kept under separate cover and sent to ethics committees and sites, as applicable.

Appendix I: Device Deficiency/Complaint Handling

The international division Event Report Form should be completed by a SJM representative or the site per country reporting timeline requirements and supplemental information (in English) attached. Device deficiencies/complaints should be emailed to SJM at: Complaints_Amplatzer@sjm.com. Please contact your local SJM representative to coordinate product returns.

EVENT REPORT FORM

INTERNATIONAL DIVISION

CATD - Amplatzer

Division Ref. No. *(Division use only)*	Return Authorization No. *(Division use only)*	Other: *(Country Office or User use only)*

DEVICE INFORMATION

Reorder or Model No.	Serial No. Lot No.	**Returning device?** ☐ Yes ☐ No ☐ Unknown
Implant Date:	Explant Date:	Date of Event:

Device under clinical investigation? ☐ ***Yes*** ☐ ***No*** — ***Was the device reused/resterilized?*** ☐ ***Yes*** ☐ ***No***

Requesting Service/Repair? ☐ Yes ☐ No

ASSOCIATED DEVICES/ACCESSORIES

Type	Model No.	Serial No. Lot No.	Implant Date	Explant Date	Returning Device? ☐ Yes ☐ No ☐ Unknown	Software Version
Type	Model No.	Serial No. Lot No.	Implant Date	Explant Date	Returning Device? ☐ Yes ☐ No ☐ Unknown	Software Version
Type	Model No.	Serial No. Lot No.	Implant Date	Explant Date	Returning Device? ☐ Yes ☐ No ☐ Unknown	Software Version
Type	Model No.	Serial No. Lot No.	Implant Date	Explant Date	Returning Device? ☐ Yes ☐ No ☐ Unknown	Software Version

EVENT

**Healthcare Professional reporting to SJM:**	**Name:**	**Title:**
**SJM employee informed of the event:**	**Name:**	**Title:**

Notification Method: — **Date reported to SJM Employee:**

Replacement Device: Reorder or Model No: — **Serial No.** — **Lot No:**

Does the Physician allege that the event was caused by the device? ☐ **Yes** ☐ **No** ☐ **Unknown**

Detailed information regarding the circumstances of the event (include information such as software version, hardware system being used, etc.):

Attach copies of all supplemental documentation to this report in English

This confidential document is the property of St. Jude Medical and shall not be reproduced, distributed, disclosed or used without the express written consent of St. Jude Medical

PATTTi:N'T or D '/1//A			
1'\ame or identifif'r:	Agl"	Sl·x:	1 v.'dght:
.MedicaJ hislory:<br>CondiHon ufkt·lWl'nt:			
Did the- pa.tient e-xperience any ad'\erse consequences due- to this event'! O Yes O No O Unknown<br>Please desn·ibe:			
\Vas the patient taking anti-coagulants as prescrib<.><J? OYes O :'lro O Unknown O 1'\/A			
l.i!'<t nthet· medicatinns:			

PHYSJCJAN /INSTITUTION / DL'>TRIBUTOR	Analysis kHl'l' t'l'tltwsll'tl: O Yl'S O 1'\o		
lnslitution Ul'Di-,;h·ibutu•·:	Custorrwr·An:.uunt Nu:		
Physician:	\.Yas the physician ce•·tified? OYes O :' In O :'11'/\.		
Address:	City:	State:	Zip:
Phonl'!	Cuuntt·)':		
Fax:	F.mail Address:		

POR,>vr sun'vfTTrrm nY	
1'\ame:	Title:
Phone:	Country:
Fax:	E-muil:
Date forwm·ded to SJ!\o1Dh·ision:	Sigllature:

Appendix J: Modified Rankin Scale (mRS)

Subject Identifier: ______________________________

Rater Name: ______________________________

Date: ______________________________

Score	Description
0	No symptoms at all
1	No significant disability despite symptoms; able to carry out all usual duties and activities
2	Slight disability; unable to carry out all previous activities, but able to look after own affairs without assistance
3	Moderate disability; requiring some help, but able to walk without assistance
4	Moderately severe disability; unable to walk without assistance and unable to attend to own bodily needs without assistance
5	Severe disability; bedridden, incontinent and requiring constant nursing care and attention
6	Dead

TOTAL (0-6): _____

Comments:

____

____

____

____

Appendix K: Intracardiac Echocardiography (ICE) Sub-Study Requirements

Requirements for intracardiac echocardiography (ICE) imaging include:

- Notification to SJM Clinical when ICE will be used for study implants:

Contact	Email	Address	Telephone/Mobile/Fax
Anne Marie Harcarik<br>Sr. Director Clinical Affairs	AHarcarik@sjm.com	LAA Occlusion<br>St. Jude Medical<br>5050 Nathan Lane North<br>Plymouth, MN 55442<br>USA	Direct +1 6517566469<br>Main +1 7635139227<br>Mobile +1 6122195601<br>Fax +1 8772572381
Sarah Gallagher<br>Clinical Programs Manager	sgallagher02@sjm.com	LAA Occlusion<br>St. Jude Medical<br>5050 Nathan Lane North<br>Plymouth, MN 55442<br>USA	Direct +1 6517566937<br>Main +1 7635139227<br>Mobile +1 6514855744<br>Fax +1 8772572381

- Minimum of 5-10 ACP or Amulet cases using ICE performed by the study implanter/investigator
- Baseline TOE LAA measurements to assess the presence of thrombus and LAA anatomy
- Combined fluoroscopic and ICE guidance during the Amulet implant procedure to confirm device positioning and deployment
- Verification by ICE or fluoroscopy to confirm device stability

Appendix L: Cardiovascular Mortality Definition

Cardiovascular Mortality	▪ Death due to proximate cardiac cause e.g. myocardial infarction, cardiac tamponade, worsening heart failure, or endocarditis<br>▪ Death caused by non-coronary, non-CNS vascular conditions such as: pulmonary embolism, ruptured aortic aneurysm, dissecting aneurysm or other vascular disease<br>▪ Death from vascular CNS causes from:<br>o hemorrhagic stroke<br>o ischemic stroke<br>▪ All procedure-related deaths including those related to a complication of the procedure or treatment for a complication of the procedure<br>▪ Sudden or unwitnessed death defined as non-traumatic, unexpected fatal event occurring within one hour of the onset of symptoms in an apparently healthy subject. If death is not witness, the definition applies when the victim was in good health 24 hours before the event<br>▪ Death of unknown cause
