# Supplementary material for: Length of stay following percutaneous left atrial appendage occlusion: Data from the prospective, multicenter Amplatzer Amulet Occluder Observational Study
Source: PLoS One. 2021 Aug 10;16(8):e0255721. doi: 10.1371/journal.pone.0255721 (PMC8354446; doi:10.1371/journal.pone.0255721)
Supplement: S2 File — (PDF) [file pone.0255721.s003.pdf]

Site Code	Site Name	Site Country	IRB Name	IRB City	IRB Zip	IRB Country
AU4839	Specialist Cardiology	AUS	Bellberry Human Research Ethics Committee	Eastwood	5063	Australia
AU1824	Sydney Adventist Hospital	AUS	Bellberry Human Research Ethics Committee	Eastwood	5063	Australia
EU2549	Hospital Universitario Son Espases	SPAIN	CEI - Islas Baleares	Palma	7010	Spain
EU2283	Hospital Universitario de Salamanca	SPAIN	CEI Clinica del Hospital Universitario de Salamanca	Salamanca	37007	Spain
ES4600	Hospital Universitario Virgen Macarena	SPAIN	CEI de los Hospitales Universitarios Virgen Macarena – Virgen del Rocío	Sevilla	41009	Spain
EU0747	Hospital Clinic I Provincial de Barcelona	SPAIN	CEIm Hospital Clinic de Barcelona	Barcelona	8036	Spain
DE3998	Universitätsmedizin Berlin - Campus Benjamin Franklin (CBF)	GERMANY	Charite Universitaetsmedizin Berlin Ethikkommission	Berlin	10117	Germany
EU3756	Azienda Ospedaliera S.Anna e S.Sebastiano	ITALY	Comitato Etico	Caserta	81100	Italy
EU2299	Centro Cardiologico Monzino	ITALY	Comitato Etico del Centro Cardiologico Monzino	Milano	20141	Italy
EU3717	Istituto Clinico Humanitas	ITALY	Comitato Etico Humanitas	Rozzano (MI)	20089	Italy
EU2169	Ospedale Luigi Sacco	ITALY	Comitato Etico Interaziendale Milano Area A	Milano	20157	Italy
EU3023	Ospedale San Raffaele	ITALY	Comitato Etico Ospedale San Raffaele	Milano		Italy
EU2376	Ospedale dell'Angelo	ITALY	Comitato etico per la Sperimentazione Clinica (CESC)	Mestre		Italy
EU1625	Università degli Studi di Padova	ITALY	Comitato Etico per la Sperimentazione Clinica della Provincia di Padova	Padova	35128	Italy
EU1905	Nuovo Ospedale Civile Sant-Agostino-Estense	ITALY	Comitato Etico Provinciale	Modena	41124	Italy
IT3932	Fondazione Toscana Gabriele Monasterio	ITALY	Comitato Etico Sperimentazione Clinica Ceavno	Pisa		Italy
CL4899	Hospital Clinico San Borja Arriarán	CHILE	Comité Científico SSMC	Santiago		Chile
EU3785	UZ Gasthuisberg	BELGIUM	Commissie Medische Ethiek UZ KU Leuven/Onderzoek	Leuven	3000	Belgium
EU0336	CHRU Lille	FRANCE	CPP Ile-de-France VI	Paris	75651	France
EU0626	CHU d'Amiens	FRANCE	CPP Ile-de-France VI	Paris	75651	France
EU1616	CHU du Bocage	FRANCE	CPP Ile-de-France VI	Paris	75651	France
EU0629	Hopital Haut Leveque	FRANCE	CPP Ile-de-France VI	Paris	75651	France

EU3 424	Hopital Henri Mondor	FRANCE	CPP Ile-de-France VI	Paris	756 51	France
EU2 115	Rigshospitalet	DENMARK	De Videnskabsetiske Komiteer for Region Hovedstaden	Hillerød	3400	Denmark
EU0 746	Skejby University Hospital	DENMARK	De Videnskabsetiske Komiteer for Region Hovedstaden	Hillerød	3400	Denmark
EU1 672	Basel University Hospital	SWTZRLD	EKNZ	Basel	4056	Switzerland
AU4 592	Epworth Hospital	AUS	Epworth HealthCare Human Research Ethics Committee	RICHMOND	2131	Australia
EU3 749	Slaskie Centrum Chorob Serca	POLAND	Ethics Commitee of Med. University of Silesia	Katowice	40-055	Poland
EU1 957	AKH - Wien	AUSTRIA	ethik kommission	Vienna	1090	Austria
EU3 450	Medizinische Einrichtungen der Universität Düsseldorf	GERMANY	Ethikkommission an der Med. Fakultät der HHU Düsseldorf	Düsseldorf	40225	Germany
EU0 170	Cardioangiologisches Centrum am Bethanien Krankenhaus	GERMANY	Ethik-Kommission bei der Landesärztekammer Hessen	Frankfurt am Main	60314	Germany
EU2 791	CardioVaskuläres Centrum St. Katharinen	GERMANY	Ethik-Kommission bei der Landesärztekammer Hessen	Frankfurt am Main	60314	Germany
DE4 097	Klinikum Frankfurt Höchst	GERMANY	Ethik-Kommission bei der Landesärztekammer Hessen	Frankfurt am Main	60314	Germany
DE4 590	Zentrum fur Herzgesundheit/Kardiologie am Alice Hospital	GERMANY	Ethik-Kommission bei der Landesärztekammer Hessen	Frankfurt am Main	60314	Germany
DE4 829	Internistisches Klinikum München SUD	GERMANY	Ethik-Kommission bei der Landesärztekammer Hessen	Frankfurt am Main	60314	Germany
EU3 292	Segeberger Kliniken GmbH	GERMANY	Ethik-Kommission bei der Landesärztekammer Hessen	Frankfurt am Main	60314	Germany
EU0 623	Klinikum Coburg GmbH	GERMANY	Ethik-Kommission bei der Medizinischen Fakultät Würzburg	Würzburg	97078	Germany
EU2 111	St. Marien-Hospital-Bonn	GERMANY	Ethikkommission d. Aerztekammer Nordrhein	Duesseldorf	40474	Germany
EU3 650	Isarherzzentrum München	GERMANY	Ethikkommission der Bayrischen Landesärztekammer	Munich	D-81677	Germany
DE4 019	Herzzentrum Dresden GmbH Universitätsklinik	GERMANY	Ethikkommission der Technischen Universitaet Dresden	Dresden	01307	Germany
EU2 426	Universitätsklinikum Ulm	GERMANY	Ethikkommission der Universitaet Ulm	Ulm	89081	Germany

EU2 820	Harzklinikum Dorothea Christiane Erxleben GmbH	GER MAN Y	Ethik-Kommission des Landes Sachsen-Anhalt	Halle (Saale)	061 08	German y
EU4 614	Universitätsklinikum Tübingen Medizinische Klinik-Kardio.	GER MAN Y	Ethikkommission Med.Fak. Univ Eberhard-Karls+Tubingen	Tübinge n	720 74	German y
EU1 874	Nemocnice Na Homolce	CZEC H R	Etická komise Nemocnice Na Homolce	Prague	150 30	Czech Republi c
EU2 579	Clínica Universidad de Navarra	SPAI N	Gobierno de Navarra	Pamplo na		Spain
AS0 116	The University of Hong Kong (Queen Mary Hospital)	HON G KO	HKU/HA HKW IRB	Hong Kong		Hong Kong
EU2 126	Hospital de la Santa Creu I Sant Pau	SPAI N	Hospital de la Santa Creu i Sant Pau	Barcelo na	080 25	Spain
EU2 103	Hospital Universitario Infanta Cristina	SPAI N	Hospital Universitario Infanta Cristina	Badajoz	060 80	Spain
BE4 627	Hôpital Civil Marie Curie	BELG IUM	ISPPC OM008	Montig ny le tilleul	611 0	Belgium
AS0 967	Prince of Wales Hospital	HON G KO	Joint CUHK-NT East Cluster CREC	Hong Kong		Hong Kong
DE4 612	Rems-Murr-Klinik Winnenden	GER MAN Y	Landesärztekammer Baden-Württemberg	Stuttgar t	705 65	German y
EU2 119	Zentralklinik Bad Berka GmbH	GER MAN Y	Landesärztekammer Thüringen Ethikkommission	Jena	077 51	German y
EU2 779	Mater Misericordiae University Hospital	IRELA ND	Mater Misericordiae University Hospital REC	Dublin		Irelan d
EU2 128	Amsterdam Academic Medical Centre (AMC)	NETH RLD	Medische Ethische Toetsingscommissie (MEC)	Amsterdam		Netherl ands
EU0 722	Karolinska University Hospital, Solna	SWE DEN	Regionala etikprövningsnämnden Stockholm	Stockho lm	171 77	Swede n
EU0 483	Sahlgrenska University Hospital - Gothenburg	SWE DEN	Regionala etikprövningsnämnden Stockholm	Stockho lm	171 77	Swede n
EU3 101	Rikshospitalet	NOR WAY	REK sor-ost	Oslo	031 8	Norwa y
AU2 300	Royal Adelaide Hospital	AUS	Royal Adelaide Hospital Human Research Ethics Committee	Adelaid e	500 0	Australi a
ME 134 7	Sheba Medical Center	ISRA EL	Sheba Medical Center Helsinki Committee	Tel Hashom er	526 21	Israel
EU3 462	Kings College Hospital	UK	South East Coast Brighton and Sussex	London	SE1 6LH	United Kingdo m
EU2 099	Liverpool NHS Trust	UK	South East Coast Brighton and Sussex	London	SE1 6LH	United Kingdo m

EU1 954	St. Thomas Hospital	UK	South East Coast Brighton and Sussex	London	SE1 6LH	United Kingdom
EU1 855	The Royal Sussex County Hospital	UK	South East Coast Brighton and Sussex	London	SE1 6LH	United Kingdom
UK4 610	University Hospital North Staffordshire	UK	South East Coast Brighton and Sussex	London	SE1 6LH	United Kingdom
EU2 924	John Radcliffe Hospital	UK	South East Coast Brighton and Sussex	London	SE1 6LH	United Kingdom
AU4 702	Fiona Stanley Hospital	AUS	South Metropolitan Health Service Human Research Ethics Committee	Murdoch	6150	Australia
PL4 195	The Cardinal Stefan Wyszynski Institute of Cardiology	POLAND	Terenowej Komisia Bioetycznej	Warszawa	04-628	Poland
EU2 005	Helsinki University Central Hospital (HYKS)	FINLAND	VSSHP Eettinen toimikunta	Turku	20521	Finland
EU1 604	Tampere University Hospital	FINLAND	VSSHP Eettinen toimikunta	Turku	20521	Finland
EU1 863	Turku University Hospital	FINLAND	VSSHP Eettinen toimikunta	Turku	20521	Finland
