# Supplementary material for: Length of stay following percutaneous left atrial appendage occlusion: Data from the prospective, multicenter Amplatzer Amulet Occluder Observational Study
Source: PLoS One. 2021 Aug 10;16(8):e0255721. doi: 10.1371/journal.pone.0255721 (PMC8354446; doi:10.1371/journal.pone.0255721)
Supplement: Attachment — Submitted filename: Response to Reviewers.docx [file pone.0255721.s004.docx]

Please include the following items when submitting your revised manuscript:

1. A rebuttal letter that responds to each point raised by the academic editor and reviewer(s). You should upload this letter as a separate file labeled 'Response to Reviewers'.
2. A marked-up copy of your manuscript that highlights changes made to the original version. You should upload this as a separate file labeled 'Revised Manuscript with Track Changes'.
3. An unmarked version of your revised paper without tracked changes. You should upload this as a separate file labeled 'Manuscript'.

If you would like to make changes to your financial disclosure, please include your updated statement in your cover letter. Guidelines for resubmitting your figure files are available below the reviewer comments at the end of this letter.

If applicable, we recommend that you deposit your laboratory protocols in [protocols.io](http://protocols.io) to enhance the reproducibility of your results. Protocols.io assigns your protocol its own identifier (DOI) so that it can be cited independently in the future. For instructions see: [http://journals.plos.org/plosone/s/submission-guidelines#loc-laboratory-protocols](https://urldefense.com/v3/__http:/journals.plos.org/plosone/s/submission-guidelines*loc-laboratory-protocols__;Iw!!BBM_p3AAtQ!bbxc8iIUWjFD2hiK_SX0RK-CzdST5pc5k2Vn0Sbo-DVOY5sk4LOtdiV9L8OMJUg_$)

We look forward to receiving your revised manuscript.

Kind regards,

Giuseppe Andò, M.D., Ph.D.
Academic Editor
PLOS ONE

Journal Requirements:

When submitting your revision, we need you to address these additional requirements.

1. Please ensure that your manuscript meets PLOS ONE's style requirements, including those for file naming. The PLOS ONE style templates can be found at

[https://journals.plos.org/plosone/s/file?id=wjVg/PLOSOne_formatting_sample_main_body.pdf](https://urldefense.com/v3/__https:/journals.plos.org/plosone/s/file?id=wjVg*PLOSOne_formatting_sample_main_body.pdf__;Lw!!BBM_p3AAtQ!bbxc8iIUWjFD2hiK_SX0RK-CzdST5pc5k2Vn0Sbo-DVOY5sk4LOtdiV9L3gmujsm$)and

[https://journals.plos.org/plosone/s/file?id=ba62/PLOSOne_formatting_sample_title_authors_affiliations.pdf](https://urldefense.com/v3/__https:/journals.plos.org/plosone/s/file?id=ba62*PLOSOne_formatting_sample_title_authors_affiliations.pdf__;Lw!!BBM_p3AAtQ!bbxc8iIUWjFD2hiK_SX0RK-CzdST5pc5k2Vn0Sbo-DVOY5sk4LOtdiV9L-ucYH5K$)

- **Reply:** We went through the PLOS ONE’s style requirements and updated the manuscript and file names accordingly.

2. Please provide additional details on the Clinical Trial study design within the Methods section and ensure that you have provided sufficient information to allow your work to be replicated.
Furthermore, thank you for providing the statement “Length of stay was not a pre-specified endpoint in the study, but we are investigating length of stay as part of a post-hoc sub-analysis. This project was presented and accepted by the scientific board.” in response to our query regarding the discrepancy in the study outcome. Please also provide this statement of clarification in the main manuscript text.

- **Reply:** We added this statement to our method section and provide the full study design as supplementary file S1. Please see p. 4 l.78-82 and supplementary file S1

3.Thank you for including your ethics statement:  "   Ethical standards: This study has been approved by the appropriate ethics committee and has therefore been performed in accordance with the ethical standards laid down in the 1964 Declaration of Helsinki and its later amendments. All persons gave their informed consent prior to their inclusion in the study. Details that might disclose the identity of the subjects under study are omitted.".

Please amend your current ethics statement to include the full name of the ethics committee/institutional review board(s) that approved your specific study.

- **Reply:** We included a supplement file with all review boards which approved the study. (Supplementary file S2)

Once you have amended this/these statement(s) in the Methods section of the manuscript, please add the same text to the “Ethics Statement” field of the submission form (via “Edit Submission”).

For additional information about PLOS ONE ethical requirements for human subjects research, please refer to [http://journals.plos.org/plosone/s/submission-guidelines#loc-human-subjects-research](https://urldefense.com/v3/__http:/journals.plos.org/plosone/s/submission-guidelines*loc-human-subjects-research__;Iw!!BBM_p3AAtQ!bbxc8iIUWjFD2hiK_SX0RK-CzdST5pc5k2Vn0Sbo-DVOY5sk4LOtdiV9L2MNJ8eH$).

4. Your ethics statement should only appear in the Methods section of your manuscript. If your ethics statement is written in any section besides the Methods, please move it to the Methods section and delete it from any other section. Please ensure that your ethics statement is included in your manuscript, as the ethics statement entered into the online submission form will not be published alongside your manuscript.

5.Thank you for stating the following in the Financial Disclosure section:

"The funders had no role in study design, data collection and analysis, decision to publish, or preparation of the manuscript."

We note that one or more of the authors are employed by a commercial company: Harzklinikum Dorothea Christiane Erxleben GmbH

1. Please provide an amended Funding Statement declaring this commercial affiliation, as well as a statement regarding the Role of Funders in your study. If the funding organization did not play a role in the study design, data collection and analysis, decision to publish, or preparation of the manuscript and only provided financial support in the form of authors' salaries and/or research materials, please review your statements relating to the author contributions, and ensure you have specifically and accurately indicated the role(s) that these authors had in your study. You can update author roles in the Author Contributions section of the online submission form.

- **Reply:** Harzklinikum Dorothea Christiane Erxleben GmbH is not a funder of the study but a commercial company. It provided support in the form of salaries for the author S.F., but did not have any additional role in the study design, data collection and analysis, decision to publish, or preparation of the manuscript

Please also include the following statement within your amended Funding Statement.

“The funder provided support in the form of salaries for authors [insert relevant initials], but did not have any additional role in the study design, data collection and analysis, decision to publish, or preparation of the manuscript. The specific roles of these authors are articulated in the ‘author contributions’ section.”

If your commercial affiliation did play a role in your study, please state and explain this role within your updated Funding Statement.

- **Reply:** The funder (Abbott) provided support in the form of salaries for authors R.G. and H.Z., but did not have any additional role in the study design, data collection and analysis, decision to publish, or preparation of the manuscript.

2. Please also provide an updated Competing Interests Statement declaring this commercial affiliation along with any other relevant declarations relating to employment, consultancy, patents, products in development, or marketed products, etc.

- **Reply:** Dr. Nielsen-Kudsk has served as a proctor for Abbott and is a consultant for Abbott and Boston Scientific. Dr. Schmidt has served as a consultant for Boston Scientific and Medtronic. Dr. Mazzone has served as a consultant for Abbott, Boston Scientific, and Medtronic. Dr. Berti has served as a proctor for Abbott. Dr. Fischer has served as a proctor for Biotronik and Boston Scientific and is a consultant for Abbott. Dr. Lund has served as a proctor for Abbott. Dr. Montorfano has served as a proctor for Abbott, Boston Scientific, and Edwards Lifesciences. Dr. Hildick-Smith has served as a proctor and consultant for Abbott. Mr. Gage and Ms. Hong Zhao are employees of Abbott (funder of the initial study). Dr. Zeus has received consulting fees, travel expenses or study honorariums from Medtronic and Edwards Lifesciences outside of this work. All other authors have nothing to disclose with regard to this project.

 Within your Competing Interests Statement, please confirm that this commercial affiliation does not alter your adherence to all PLOS ONE policies on sharing data and materials by including the following statement: "This does not alter our adherence to  PLOS ONE policies on sharing data and materials.” (as detailed online in our guide for authorshttp://journals.plos.org/plosone/s/competing-interests) . If this adherence statement is not accurate and  there are restrictions on sharing of data and/or materials, please state these. Please note that we cannot proceed with consideration of your article until this information has been declared.

- **Reply:** No data are available on a public platform. Individual deidentified participant data cannot be shared. The sponsor of this study (Abbott) is unable to legally distribute/provide access to the study data at the time of publication due to privacy concern.  However, Abbott provides contact information instead where an interested researcher could apply to gain access to the data

Website: <https://www.cardiovascular.abbott/us/en/hcp/investigator-sponsored-studies.html>

Email: [abbottissrequests@abbott.com](mailto:abbottissrequests@abbott.com)

 Please include both an updated Funding Statement and Competing Interests Statement in your cover letter. We will change the online submission form on your behalf.

- **Reply:** We did include an updated Funding and Competing Interest Statement in our cover letter.

Please know it is PLOS ONE policy for corresponding authors to declare, on behalf of all authors, all potential competing interests for the purposes of transparency. PLOS defines a competing interest as anything that interferes with, or could reasonably be perceived as interfering with, the full and objective presentation, peer review, editorial decision-making, or publication of research or non-research articles submitted to one of the journals. Competing interests can be financial or non-financial, professional, or personal. Competing interests can arise in relationship to an organization or another person. Please follow this link to our website for more details on competing interests: http://journals.plos.org/plosone/s/competing-interests

6. We note that you have indicated that data from this study are available upon request. PLOS only allows data to be available upon request if there are legal or ethical restrictions on sharing data publicly. For more information on unacceptable data access restrictions, please see [http://journals.plos.org/plosone/s/data-availability#loc-unacceptable-data-access-restrictions](https://urldefense.com/v3/__http:/journals.plos.org/plosone/s/data-availability*loc-unacceptable-data-access-restrictions__;Iw!!BBM_p3AAtQ!bbxc8iIUWjFD2hiK_SX0RK-CzdST5pc5k2Vn0Sbo-DVOY5sk4LOtdiV9LyerWFqL$).

- **Reply:** No data are available on a public platform. Individual deidentified participant data cannot be shared. The sponsor of this study (Abbott) is unable to legally distribute/provide access to the study data at the time of publication due to privacy concern.  However, Abbott provides contact information instead where an interested researcher could apply to gain access to the data

Website: <https://www.cardiovascular.abbott/us/en/hcp/investigator-sponsored-studies.html>

Email: [abbottissrequests@abbott.com](mailto:abbottissrequests@abbott.com)

In your revised cover letter, please address the following prompts:

a) If there are ethical or legal restrictions on sharing a de-identified data set, please explain them in detail (e.g., data contain potentially sensitive information, data are owned by a third-party organization, etc.) and who has imposed them (e.g., an ethics committee). Please also provide contact information for a data access committee, ethics committee, or other institutional body to which data requests may be sent.

b) If there are no restrictions, please upload the minimal anonymized data set necessary to replicate your study findings as either Supporting Information files or to a stable, public repository and provide us with the relevant URLs, DOIs, or accession numbers. For a list of acceptable repositories, please see [http://journals.plos.org/plosone/s/data-availability#loc-recommended-repositories](https://urldefense.com/v3/__http:/journals.plos.org/plosone/s/data-availability*loc-recommended-repositories__;Iw!!BBM_p3AAtQ!bbxc8iIUWjFD2hiK_SX0RK-CzdST5pc5k2Vn0Sbo-DVOY5sk4LOtdiV9L0mtmsyX$).

We will update your Data Availability statement on your behalf to reflect the information you provide.

- **Reply:** Thank you. An updated version of the data availability statement can be found in the cover letter.

7.We note that [Figure(s) 3] in your submission contain map images which may be copyrighted. All PLOS content is published under the Creative Commons Attribution License (CC BY 4.0), which means that the manuscript, images, and Supporting Information files will be freely available online, and any third party is permitted to access, download, copy, distribute, and use these materials in any way, even commercially, with proper attribution. For these reasons, we cannot publish previously copyrighted maps or satellite images created using proprietary data, such as Google software (Google Maps, Street View, and Earth). For more information, see our copyright guidelines: [http://journals.plos.org/plosone/s/licenses-and-copyright](https://urldefense.com/v3/__http:/journals.plos.org/plosone/s/licenses-and-copyright__;!!BBM_p3AAtQ!bbxc8iIUWjFD2hiK_SX0RK-CzdST5pc5k2Vn0Sbo-DVOY5sk4LOtdiV9L9Um2ZqV$).

We require you to either (1) present written permission from the copyright holder to publish these figures specifically under the CC BY 4.0 license, or (2) remove the figures from your submission:

- **Reply:** We decided to remove Fig 3.

8. Please include captions for your Supporting Information files at the end of your manuscript, and update any in-text citations to match accordingly. Please see our Supporting Information guidelines for more information: http://journals.plos.org/plosone/s/supporting-information.

Reviewers' comments:

Reviewer's Responses to Questions

**Comments to the Author**

1. Is the manuscript technically sound, and do the data support the conclusions?

The manuscript must describe a technically sound piece of scientific research with data that supports the conclusions. Experiments must have been conducted rigorously, with appropriate controls, replication, and sample sizes. The conclusions must be drawn appropriately based on the data presented.

Reviewer #1: Yes

Reviewer #2: Partly

Reviewer #3: Yes

2. Has the statistical analysis been performed appropriately and rigorously?

Reviewer #1: Yes

Reviewer #2: No

Reviewer #3: Yes

3. Have the authors made all data underlying the findings in their manuscript fully available?

The [PLOS Data policy](https://urldefense.com/v3/__http:/www.plosone.org/static/policies.action*sharing__;Iw!!BBM_p3AAtQ!bbxc8iIUWjFD2hiK_SX0RK-CzdST5pc5k2Vn0Sbo-DVOY5sk4LOtdiV9L-Y_8JeV$) requires authors to make all data underlying the findings described in their manuscript fully available without restriction, with rare exception (please refer to the Data Availability Statement in the manuscript PDF file). The data should be provided as part of the manuscript or its supporting information, or deposited to a public repository. For example, in addition to summary statistics, the data points behind means, medians and variance measures should be available. If there are restrictions on publicly sharing data—e.g. participant privacy or use of data from a third party—those must be specified.

Reviewer #1: Yes

Reviewer #2: No

Reviewer #3: Yes

4. Is the manuscript presented in an intelligible fashion and written in standard English?

PLOS ONE does not copyedit accepted manuscripts, so the language in submitted articles must be clear, correct, and unambiguous. Any typographical or grammatical errors should be corrected at revision, so please note any specific errors here.

Reviewer #1: Yes

Reviewer #2: Yes

Reviewer #3: Yes

5. Review Comments to the Author

Please use the space provided to explain your answers to the questions above. You may also include additional comments for the author, including concerns about dual publication, research ethics, or publication ethics. (Please upload your review as an attachment if it exceeds 20,000 characters)

Reviewer #1: Please revise the statement in the Introduction to the effect that there are randomized data showing that left atrial appendage occlusion (LAAO) is preferable to oral anticoagulation (OAC) in patients without contraindication to oral anticoagulation. There was a mortality benefit in PROTECT-AF at long-term. You may then add that illogically general customs and guidelines recommend the use of LAAO in patients with AF only in those who are unable to tolerate long-term anticoagulation use due to contraindications, high bleeding risk in general, low drug tolerance, or low drug adherence.

- **Reply:** This is an important comment. In the introduction we stated that “However, percutaneous left atrial appendage occlusion (LAAO) has emerged as a favorable non-pharmacological stroke prevention strategy in patients with AF who are unable to tolerate long-term anticoagulation use due to contraindications, high bleeding risk in general, low drug tolerance or low drug adherence”. We checked again that we did not emphasize that LAAO is preferable to OAC in patients without contraindications to oral anticoagulation.

The main difference lies between same day procedures and procedures with overnight surveillance. Please make a separate group of "Same day discharge" patients. It is, of course, not important for this study if patients spent the night before the procedure at the hospital or not.
So, your new groups should be: Same day discharge (or Outpatient procedure, meaning 0 night after LAAO; according to Figure 2, there were 62 such patients), early discharge (1 night), Regular discharge (2/3 nights), and Late discharge (≥4 nights).

- **Reply:** This was a crucial comment and we discussed its implications thoroughly. According to the reviewers’ recommendation we did rearrange the grouping in patients with same-day discharge, early discharge, regular discharge and late discharge. Results are presented within the text, figures and tables accordingly throughout the whole manuscript.

This will unfortunately entail to change most statistics, tables, and figures.
What do you mean by "12 patients had no discharge visit and were excluded"? Did they die in the hospital or do you simply not know how long they stayed?

- **Reply:** Thank you for the opportunity to clarify. We went back to the original data and identified the reasons for exclusion: 8 patients had unsuccessful implant attempts (no SAEs), three died prior to discharge, and one was withdrawn after device embolization and surgical LAA ligation. We added this information in our result section and to Fig. 1. Please see Fig. 1 and p. 6 l. 128-130

In table 2, there are no figures for Intracardiac echocardiography. Looking at Total echocardiography and Transesophageal echocardiography, they seem to account for the difference, i.e., the majority of patients.

- **Reply:** The numbers for intracardiac echocardiography are now implemented in the table. Please see Table 2 (p.8)

Please provide a Table 4 with specific complications (e.g., cerebral ischemia, myocardial infarction, pericardial bleed, cardiac tamponade, device embolization, puncture site problem, bronchial aspiration or other problems of transesophageal echocardiography, pneumothorax due to central line, etc.).

- **Reply:** Thank you for this helpful comment. We added an additional table to the manuscript and report relevant adverse events related to the LAAO within this study. We embedded this information into Table 2 (p.8-9) after the SAE reporting section.

|  | **Same Day** | **Early** | **Regular** | **Late** |
| --- | --- | --- | --- | --- |
|  | **n=62** | **n=526** | **n=338** | **n=150** |
| Ischemic stroke | 0.0% | 0.0% | 0.0% | 2.0% |
| Myocardial infarction | 0.0% | 0.0% | 0.0% | 0.0% |
| Pericardial effusion without tamponade | 0.0% | 0.0% | 0.9% | 2.0% |
| Pericardial effusion with tamponade | 1.6% | 0.4% | 0.0% | 4.0% |
| Device embolization | 0.0% | 0.0% | 0.0% | 0.7% |
| Vascular access site complication | 0.0% | 0.4% | 0.6% | 5.3% |
| TEE-related complication | 1.6% | 0.0% | 0.0% | 0.7% |
| Penumothorax | 0.0% | 0.0% | 0.0% | 0.0% |

At the end, please come up with some recommendations what supports same day discharge and what mandates at least 1 night at the hospital after LAAO (e.g., chest pain, pericardial effusion at echocardiography, etc.). So, readers can benefit from the paper.

- **Reply:** Looking at the provided data, this is a tricky question. We do not see a relevant difference in complications during the same day and early discharge group. Therefore it seems reasonable to base the decision upon whether same day or early discharge is performed on patient wish and social environment. Obviously a successful procedure without any complication is a precondition for both time frames (p. 14 l. 269-273).

Reviewer #2: The manuscript addresses an interesting topic. The data have some specific features deserving to be analyzed. The research questions may lead to improvements in the existing literature. Nevertheless, there are several methodological/statistical issues which must be addressed to ensure the reliability of the results.

1. The data are not fully available. This is not in line with the journal's guidelines ([https://journals.plos.org/plosone/s/data-availability](https://urldefense.com/v3/__https:/journals.plos.org/plosone/s/data-availability__;!!BBM_p3AAtQ!bbxc8iIUWjFD2hiK_SX0RK-CzdST5pc5k2Vn0Sbo-DVOY5sk4LOtdiV9L3BtH-dL$)) and does not allow the reviewer to reproduce the results. Please, upload the data and the code used to obtain the results.

- **Reply:** After discussion with the sponsor, no data are available on a public platform. Individual deidentified participant data cannot be shared. The sponsor of this study (Abbott) is unable to legally distribute/provide access to the study data at the time of publication due to privacy concern.  However, Abbott provides contact information instead where an interested researcher could apply to gain access to the data

Website: <https://www.cardiovascular.abbott/us/en/hcp/investigator-sponsored-studies.html>

Email: [abbottissrequests@abbott.com](mailto:abbottissrequests@abbott.com)

We updated our data sharing agreement accordingly.

2. The statistical methods employed should be better described. At first glance, a linear regression model is considered to model the length of stay (LOS). However, the LOS is a generally a count variable; isn't it? As counts, the correct starting point model is the Poisson regression. Please, be aware that any models is based on some assumptions which must be fulfilled to ensure the reliability of the results. For example, the linear regression must satisfy the Gauss-Markov assumptions, the Poisson regression is based on the equidispersion assumption, etc. Accordingly, please, amend the regression part choosing for the regression model according to the nature and characteristics of the dependent variable at hand.

- **Reply:** We thank the reviewer for the suggestion. We agree that Poisson regression is the appropriate model for the count data, in general. However, a limitation of the model is the assumption that the variance = the mean, which may not always be true. For the “length of stay” variable, values range from 0 to 57 days, with much larger variance (15.8) compared to the mean (2.4), indicating overdispersion. Therefore, we reanalyzed the data using a multinomial logistic model, where “length of stay” is categorized into 4 categories: 0 day (“same day discharge”), 1 day, 2-3 days, and ≥4 days as suggested by another reviewer (Reviewer #1) for meaningful clinical interpretation.

Univariate multinomial logistic analysis only identified baseline HAS-BLED score as an independent influencing factor (p=0.04, please find details Below).  Results for sex (Female vs Male) and CHA_2_DS_2_-VASc score are not statistically significant (p= 0.1 and 0.07, respectively). As shown in Table below, the odds ratio for late discharge relative to early discharge is 1.271 based on a one unit increase in HAS-BLED score, indicating an association between higher HAS-BLED score and longer length of stay following the procedure. These results were updated in the Regression Section of the manuscript accordingly. Please see p. 10 l. 181-189 and the added paragraph in the discussion section (p. 13-14 l. 254-268)

Table 3: Results of Multinomial Logistic Analysis of Length of Stay with HAS-BLED Score (please see p.10/11)

| **Odds Ratio Estimates** | | | | |
| --- | --- | --- | --- | --- |
| Effect | Length of Stay | Point Estimate | 95% Wald Confidence Limits | |
| HAS-BLED Score | Late Discharge | 1.271 | 1.077 | 1.499 |
| HAS-BLED Score | Regular Discharge | 1.032 | 0.910 | 1.172 |
| HAS-BLED Score | Same Day Discharge | 1.063 | 0.834 | 1.354 |

3. The study is multicenter and, as such, observations belonging to the same center may be correlated. In other words, the data structure is hierarchical. This hierarchy should be accounted for in the modelling, i.e. heterogeneity across centers must be addressed.

- **Reply:** Thanks for the suggestion. A total of 61 centers enrolled subjects. Among those, 14 centers enrolled 5 or less subjects with a total of 44 subjects; 13 centers enrolled between 6 and 10 subjects with a total of 101 subjects.

Therefore, a hierarchical model adding center effects may not be appropriate here due to a large number of centers and the sparseness of the data. We added this information to our method section. Please see p. 5 l. 112-114

4. A more detailed survival analysis would be appreciated. A proportional hazard Cox model may be considered (again, please, check for model's assumptions) and some comments on the main variables affecting mortality may be discussed.

- **Reply:** Thank you for this comment. We evaluated the Cox regression model, however, the proportional hazard assumption was not met. Therefore, a Cox regression model analysis was deemed not appropriate. Therefore, we did not change the survival analysis.

5. Please, provide evidence that the assumptions behind the parametric tests implemented to obtain results in Table 1 are fulfilled. If they are not, please, move to non-parametric testing procedures.

- **Reply:** Within the “Statistical analysis” sub-section of the Methods section we state the statistical method utilized in Table 1: “The Kruskal-Wallis test was used to identify differences in continuous variables between the early, regular and late discharge group. Fischer’s Exact Test was used to identify differences in categorical variables”. Therefore, this methodology utilized non-parametric testing in Table 1.

Reviewer #3: The article "Length of stay following percutaneous LAAO: data from the prospective, multicenter Amplatzer Amulet Occluder Observational Study", reports data regarding the influence of LAAO (with Amplatzer Amulet) on lenght of in-hospital stay, in a population of patients treated between 2015 and 2016.
The manuscript is well written in good English, data are elegantly analyzed and reported by the Authors.
Limitations are well described in the dedicate session.
Minor point:
- Manuscript preparation should be revised, since Figure legends are included in the manuscript (rather than listed at the end), making article revision more difficult.
Major point:

- **Reply:** Thank you for pointing this out. We went through the PLOS One style requirements and formatted the manuscript accordingly.

- The Study refers to a "early" experience with percutaneous LAAO (2015-2016) with a single "first-generation" device; this should be discussed in either positive or negative way (considering the results), due to the influence of procedural experience on both ischemic and hemorragic adverse events.

- **Reply:** We added a paragraph to the discussion to the discussion section to emphasize the effect of early generation devices on LAAO outcomes. Please see p. 12 l. 235-236 and p. 14 l. 280-282.
