# Supplementary material for: Length of stay following percutaneous left atrial appendage occlusion: Data from the prospective, multicenter Amplatzer Amulet Occluder Observational Study
Source: PLoS One. 2021 Aug 10;16(8):e0255721. doi: 10.1371/journal.pone.0255721 (PMC8354446; doi:10.1371/journal.pone.0255721)
Supplement: Attachment — Submitted filename: Response to reviewers.docx [file pone.0255721.s005.docx]

**Response to reviewers**

Reviewer #1: The amendments are in part adequate. Yet, you need to dwell more on your new group of same day discharge patients which is the most interesting group of all.

Moreover, you completely misread my initial comment. Your answer is the opposite of what I expected and intended to achieve. Please read my statement more carefully and amend your text accordingly: After 2 randomized trials showing a mortality benefit of left atrial appendage closure (LAAC) over oral anticoagulation (OAC) which materializes after 2 years and becomes more and more conspicuous with time, you cannot (cannot!) state that "However, percutaneous left atrial appendage occlusion (LAAO) has emerged as a favorable non pharmacological stroke prevention strategy in patients with AF who are unable to tolerate long-term anticoagulation use due to contraindications, high bleeding risk in general, low drug tolerance or low drug adherence(6)". You have to critizise that this admittedly still predominant behavior makes no sense and must be changed in practice and in the guidelines. LAAC must be the first line treatment and OAC the treatment for those who cannot have LAAC or only have less than 2 years of life expectancy. LAAC has an initial risk of a severe problem of about 3-5% but then lacks the annual risk of a severe bleeding of about 2% germane to OAC. Equipoise in risk can be expected after 2 years. Thereafter, LAAC accumulates an ever growing advantage. Please change the respective statements in Introduction and Discussion.

- **Reply:** We thank the reviewer for his clear statement on the benefit of LAAO in patients with atrial fibrillation. In our introduction, we tried to fit in current guideline recommendations and underlined the growing evidence of further benefits of LAAO, which are not yet reflected in those recommendations. (p. 3 l- 60-70)

According to the guidelines a long-term anticoagulative strategy remains the first line therapy for stroke prevention with a Class I Level A recommendation, despite evident side effects and limited drug adherence.(5, 6) However, percutaneous left atrial appendage occlusion (LAAO) has emerged as a favorable non-pharmacological stroke prevention. After long term follow-up several randomized trials showed reductions in major bleeding, hemorrhagic stroke, and mortality as compared to warfarin (7). Despite positive clinical outcomes in trials of subjects tolerant of long-term anticoagulation, LAAO is currently only recommended for patients with AF who are unable to tolerate long-term anticoagulation use due to contraindications, high bleeding risk in general, low drug tolerance or low drug adherence(8) at a Class IIb Level B recommendation (6).

When mentioning that 12 patients were excluded because they had no discharge visit, you have to specify what that means. So, I suggest to change the sentence to something like: "Twelve patients were excluded for reasons explained under Results."

- **Reply:** We thank the reviewer for the suggestion and have updated the Figure 1 legend accordingly.

In your new group of 62 same day discharge patients, you have 1 patient each with pericardial effusion without tamponade and 1 with pericardial effusion with tamponade on the first day (their only day after the procedure and their day of discharge). Did you really discharge these patients the same day and, if yes, what happened to them?

- **Reply:** This is a very precise observation and we checked our files again. In reviewing the analysis the 1 Same Day Discharge patient with a pericardial effusion without tamponade was a transcription error into the manuscript. This value should instead be 0.0% and has been corrected in the revised manuscript. Hospital records were reviewed for the 1 Same Day Discharge patient with a pericardial effusion resulting in tamponade. By reviewing the hospital records it became apparent that the patient was discharged on day 5 post-LAAO as opposed to the same day (i.e., day 0) discharge provided by site-submitted data to the study database. Therefore, this patient‘s length of stay was updated to 5 days and the patient was recategorized from the Same Day Discharge to the Late Discharge group in the revised manuscript.

You also had 1 patient with a TEE related complication in that group. What was it and was the patient really discharged the same day?

- **Reply:** Hospital records were reviewed for this adverse event. Shortly after the LAAO intervention bleeding from the nasopharynx / upper palate was observed. The source of bleeding was identified as a lesion in the upper palate, which stopped after a local injection of Suprarenin. An additional diffuse mucous membrane nasal bleed was controlled with insertion of a tamponade. Moreover the in-patient recovery was complicated by respiratory distress and a febrile infection requiring antibiotic therapy. These complications resulted in the subject actually having a 21 day in-hospital stay following LAAO, as opposed to the same day discharge indicated by site-submitted data to the study database. Therefore, this patient‘s length of stay was updated to 21 days and the patient recategorized from the Same Day Discharge to the Late Discharge group in the revised manuscript.

All calculations were updated throughout the manuscript and figures updated accordingly.

These patients are not in keeping with your statement: "Obviously a successful procedure without any SAE is a precondition for both time frames." which not only refers to the same day discharge patients but also to the next day discharge patients.

- **Reply:** We agree with the reviewer that using the term „precondition“ is too strong. We have reworded this text to reflect the extremely high degree of patients with successful procedures (same day= 100%; early discharge = 99.8%) and low degree of patients with procedure/device related SAEs prior to discharge (same day= 0%; early discharge= 1.0%). The text now reads: A successful procedure without any SAE often occurred for patients discharged within either timeframe and may be the primary factor for allowing same day or early discharge.

Reviewer #3: The authors have deeply revised the manuscript, addressing all the Reviewer's questions and Editorial requests.

- **Reply:** We thank Reviewer #3 for their feedback leading to an improved manuscript through the revision process.
